# Generation of Novel Natural Products by Disrupting Azaphilone Synthesis in *Penicillum sclerotiorum* E23Y-1A

**DOI:** 10.3390/md24030095

**Published:** 2026-02-27

**Authors:** Wenjun Chang, Yanhua Yang, Ruijun Duan, Heye Qin, Shiwen Chen, Yanbo Zeng

**Affiliations:** 1 Hainan Provincial Key Laboratory for Functional Components Research and Utilization of Marine Bio-Resources, Institute of Tropical Bioscience and Biotechnology, Chinese Academy of Tropical Agricultural Sciences & Key Laboratory for Biology and Genetic Resources of Tropical Crops of Hainan Province, Hainan Institute for Tropical Agricultural Resources, Haikou 571101, China; changwenjun@catasitbb.cn (W.C.); duanruijun@catasitbb.cn (R.D.); qinheye@catasitbb.cn (H.Q.); chenshiwen@catasitbb.cn (S.C.); 2 School of Pharmacy and Bioengineering, Chongqing University of Technology, Chongqing 400054, China; yangyanhua1250@163.com; 3 Hainan Key Laboratory for Biosafety Monitoring and Molecular Breeding in Off-Season Reproduction Regions, Key Laboratory of Biology and Genetic Resources of Tropical Crops, Sanya Research Institute of Chinese Academy of Tropical Agricultural Sciences, Sanya 572025, China

**Keywords:** *Penicillium sclerotiorum*, azaphilone, gene knockout, metabolic shunting, novel natural products, meroterpenoid, antimicrobial activity, antitumor activity

## Abstract

Marine-derived filamentous fungi are a rich source of structurally diverse and biologically active natural products. However, many biosynthetic gene clusters (BGCs) in fungi remain silent under standard conditions. In this study, we employed a metabolic shunting strategy to disrupt azaphilone biosynthesis in the marine-derived fungus *Penicillium sclerotiorum* E23Y-1A by deleting the pathway-specific regulator gene *A00667*. HPLC analysis revealed the emergence of new metabolite peaks in the mutant strain Δ667 compared to the wild type. Subsequent purification yielded seven compounds: the mutant produced two novel meroterpenoids sclerotilins A and B (**1** and **2**) along with the known steroids ergosta-5,7,22-trien-3*β*-ol (**3**) and cerevisterol (**4**), while the wild type yielded the known steroid (22*E*)-5α,8α-epidioxyergosta-6,22-dien-3*β*-ol (**5**) and two azaphilones geumsanol G (**6**) and 5-chloro-3-[(1*E*,3*R*,4*R*,5*S*)-3,4-dihydroxy-3,5-dimethyl-1-hepten-1-yl]-1,7,8,8a-tetrahydro-7,8-dihydroxy-7-methyl-(7*R*,8*R*,8a*S*)-6*H*-2-benzopyran-6-one (**7**). Bioactivity assays showed that compound **6** exhibited moderate antimicrobial activity against *Staphylococcus aureus*, and compound **3** displayed moderate cytotoxicity against five human cancer cell lines. These results demonstrate that *A00667* is essential for azaphilone biosynthesis and that its disruption leads to the production of structurally distinct natural products, highlighting the potential of pathway engineering to redirect fungal metabolism to yield novel natural products.

## 1. Introduction

The marine environment is an invaluable source of novel natural products characterized by their structural diversity, significant bioactivity, and chemical novelty [1,2,3]. It has been reported that various filamentous fungi of marine origin, such as *Aspergillus*, *Penicillium*, and *Fusarium*, are capable of synthesizing a range of bioactive compounds that are pharmaceutically promising [4,5,6,7].

One major challenge in natural product research is the ability to discover novel, biologically active compounds. Advances in genome sequencing and biosynthetic gene cluster (BGC) prediction have revealed that fungi harbor millions of cryptic BGCs, underscoring their immense potential to produce novel natural products [8]. Diverse strategies including One Strain Many Compounds (OSMAC), cocultivation, and genetic, epigenetic, and metabolic engineering techniques are now being used to unlock their potential for producing novel natural products [9,10,11,12,13]. Metabolic shunting can be controlled through enzymatic activity, which allows researchers to engineer microorganisms by introducing or modifying genes for the production of pharmaceuticals or other valuable compounds [13,14].

*Penicillium sclerotiorum* E23Y-1A is a filamentous fungus isolated from the marine sponge *Holoxea* sp. [15]. In our previous studies, we found that azaphilones constitute the primary metabolic composition of *P. sclerotiorum*, leading to the identification of dozens of novel azaphilone compounds [15,16]. To explore its biosynthetic potential beyond azaphilone production, in this work, we implemented a metabolic shunting strategy by deleting a key regulatory gene involved in the azaphilone biosynthetic pathway. This intervention successfully suppressed azaphilone biosynthesis and enabled the discovery of new metabolites. We achieved the characterization of novel natural products while also confirming gene *A00667* as a pathway-specific regulator essential for azaphilone biosynthesis.

## 2. Results

### 2.1. The P. sclerotiorum Genome and Azaphilone BGC

**Figure 1:**
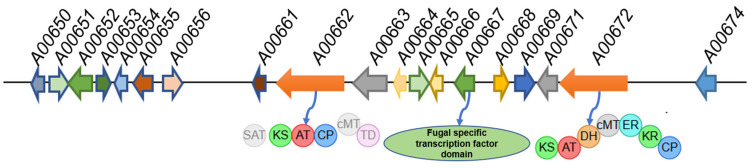
Schematic representation of azaphilone biosynthetic gene cluster in *P. sclerotiorum* E23Y-1A.

The genome of *P. sclerotiorum* strain E23Y-1A was assembled into 181 contigs, totaling 35.9 Mbp with a GC content of 48.48% and 10,868 genes predicted. Genome mining of *P. sclerotiorum* with antiSMASH (version 8.0.4) revealed 70 biosynthetic gene clusters (BGCs), dominated by 29 nonribosomal peptide synthetases (NRPSs), 20 polyketide synthases (PKSs, 8 of which were hybrids), and 12 terpene synthases. BLAST (version 2.17.0) analysis revealed that a PKS/terpene hybrid cluster (cluster 12.4), located on scaffold 12 of the *P. sclerotiorum* genome, exhibits homology to several known azaphilone biosynthetic gene clusters responsible for the biosynthesis of azasperpyranones A–F, asperfuranone, and chaetoviridin E [17,18,19,20]. The 12.4 cluster harbors two core biosynthetic genes encoding a highly reducing polyketide synthase (HRPKS, GenBank PX533551) and a non-reducing polyketide synthase (NRPKS, GenBank PX533544, Figure 1, Table S1). The genetic architecture comprising both HRPKS and NRPKS is a hallmark of many characterized azaphilone biosynthetic pathways, such as those for asperfuranone, chaetoviridin and, azanigerone [18,19,21]. The NRPKS protein A00662, which comprises starter unit acyl-CoA transacylase (SAT), *β*-ketoacyl synthase, acyltransferase, acyl carrier protein, and a terminal methyltransferase domain, exhibits 93% identity to Psc_Aza_B and 66% identity to CazM (Figure 1). These two homologs are, respectively, involved in the biosynthesis of halogenated azaphilones in the termite-symbiotic fungus *P. sclerotiorum* SNB-CN111 and in the chaetoviridin biosynthetic pathway [19,22]. Meanwhile, the HRPKS A00672 was identified to feature a seven-domain organization typical of HRPKSs, comprising *β*-ketoacyl synthase, acyltransferase, dehydratase, methyltransferase, enoylreductase, keto-reductase, and acyl carrier protein (Figure 1). A BLAST search revealed that its homologs include Psc_Aza_A (94% identity), a key azaphilone biosynthetic protein in *P. sclerotiorum* SNB-CN111 [22], and ATEG_07659 (67.0% identity), a protein within the azasperpyranone A biosynthetic cluster [17]. In addition to the core PKS genes, the 12.4 BGC also encodes other key enzymes for azaphilone biosynthesis, such as an FAD-dependent monooxygenase (A00668, GenBank PX533549) and a halogenase (A00669, GenBank PX533550), which identifies it as the putative azaphilone-producing cluster in *P. sclerotiorum* E23Y-1A (Figure 1, Table S1). The 12.4 cluster harbors two fungal-specific transcription factors, A00652 and *A00667* (GenBank PX533548), which contain a TFMHR domain predominantly found in Aspergillus and Penicillium species (Figure 1, Table S1). BLAST analysis revealed that both proteins share similarity with Zn(II)_2_Cys_6_-type transcription factors, suggesting their regulatory role in the azaphilone biosynthetic pathway of *P. sclerotiorum*. Consequently, we targeted *A00667* for gene deletion in this study to assess its necessity for azaphilone production in *P. sclerotiorum*.

### 2.2. Generation and Confirmation of Mutant Strain Δ667

**Figure 2:**
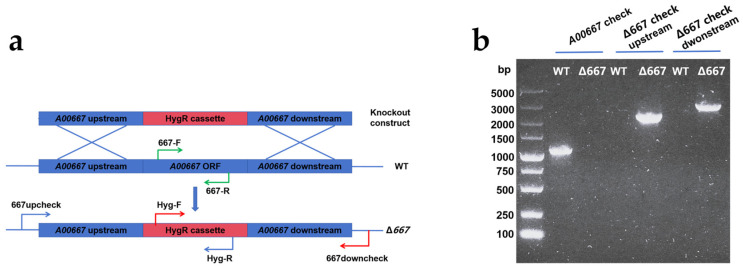
*A00667* disruption in *P. sclerotiorum* E23Y-1A. (**a**) Schematic diagram of generation of Δ667 by homologous recombination; (**b**) PCR confirmation of Δ667.

The Δ667 mutants were generated by replacing the *A00667* gene in wild-type *P. sclerotiorum* with a hygromycin resistance (HygR) cassette via homologous recombination, selected on hygromycin-containing plates, and confirmed by PCR (Figure 2). No specific 1087 bp band corresponding to the wild-type *A00667* gene was amplified in the Δ667 strain, indicating successful gene disruption (Figure 2b). Furthermore, to verify the correct integration at the target locus, the upstream and downstream junctions were amplified using primer pairs 667upcheck/Hyg-R and Hyg-F/667downcheck, respectively (Figure 2a). This yielded the expected 2254 bp and 3035 bp fragments from the Δ667 genomic DNA (Figure 2b). Collectively, these results confirm that the *A00667* gene was successfully replaced at the correct chromosomal locus.

### 2.3. Effect of Gene A00667 Disruption on Metabolite Production

**Figure 3:**
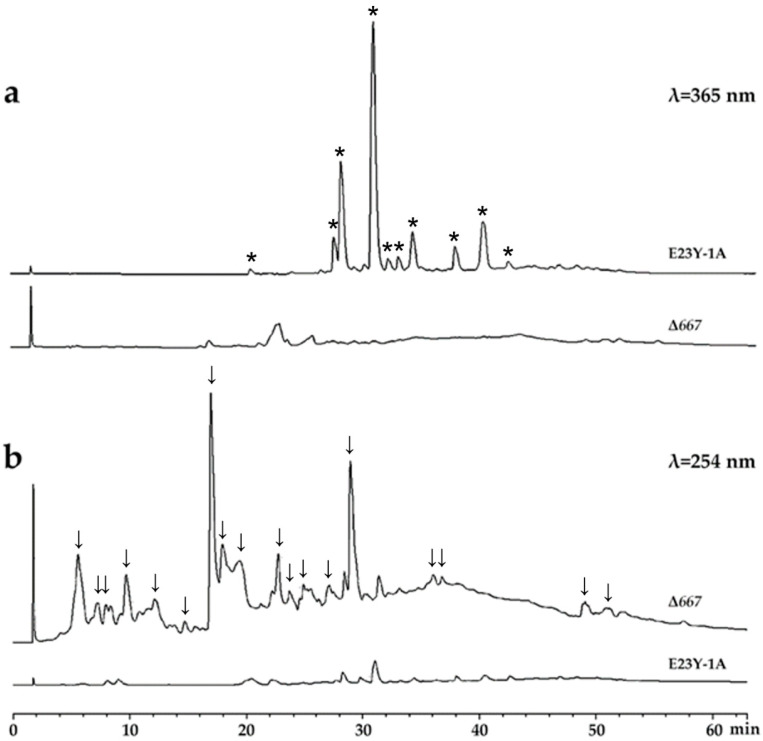
HPLC analysis of crude extracts from wild-type strain E23Y-1A and mutant strain Δ667 at 365 nm (**a**) and 254 nm (**b**). All dominant peaks, which correspond primarily to azaphilone compounds and are exclusive to wild-type strain, are labeled with asterisks (*), while newly emerged metabolite peaks unique to Δ667 mutant strain (absent in wild type) are labeled with arrows (↓).

To investigate the metabolic changes resulting from *A00667* disruption, we performed HPLC analysis on crude extracts from both the wild-type strain E23Y-1A and the mutant strain Δ667. The results showed that all dominant peaks present in the wild-type strain E23Y-1A were absent in the mutant strain Δ667 when detected at 365 nm (Figure 3a). Building on our prior work and the metabolic characterization performed in this study, we confirmed that these peaks correspond predominantly to azaphilones in E23Y-1A [15,16,23], indicating that the knockout of gene *A00667* severely disrupted the biosynthesis of azaphilone compounds. In contrast, when detected at 254 nm, a set of new peaks eluting between 0 and 30 min emerged in the HPLC profile of Δ667 compared to that of E23Y-1A, suggesting the production of novel compounds in the mutant strain (Figure 3b). These results clearly demonstrate that *A00667* is involved in the biosynthesis regulation of azaphilone compounds and that its disruption led to the production of several novel metabolites.

### 2.4. Structural Elucidation of New Compounds

**Figure 4:**
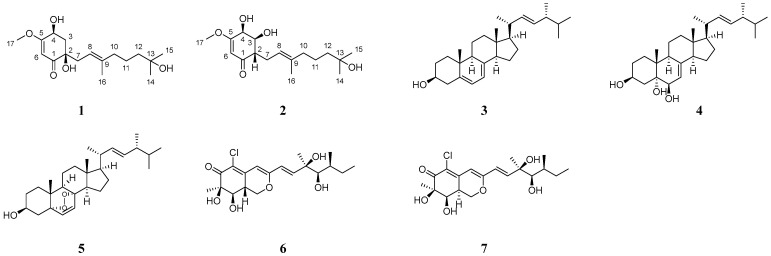
Structures of compounds.

To compare the chemical composition between the wild-type strain E23Y-1A and the mutant strain Δ667, crude extracts from both strains were purified by silica gel chromatography and semi-preparative HPLC. This purification afforded seven compounds (Figure 4), comprising two novel meroterpenoids sclerotilins A and B (**1** and **2**) [24,25] and two known steroids—ergosta-5,7,22-trien-3*β*-ol (**3**) [26,27] and cerevisterol (**4**) [28,29]—from the mutant strain 667, as well as three known compounds from the wild-type strain E23Y-1A: the steroid (22*E*)-5*α*,8*α*-epidioxyergosta-6,22-dien-3*β*-ol (**5**) [30,31], along with two azaphilones, geumsanol G (**6**) [32,33] and 5-chloro-3-[(1*E*,3*R*,4*R*,5*S*)-3,4-dihydroxy-3,5-dimethyl-1-hepten-1-yl]-1,7,8,8a-tetrahydro-7,8-dihydroxy-7-methyl-(7*R*,8*R*,8a*S*)-6*H*-2-benzopyran-6-one (**7**) [34,35]. The structures of the known compounds were confirmed by comparing their 1D/2D NMR spectroscopic data with those reported in the literature.

**Figure 5:**
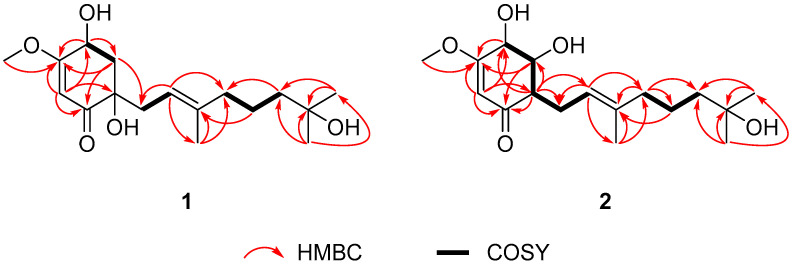
^1^H-^1^H COSY and key HMBC correlations of compounds **1** and **2**.

**Figure 6:**
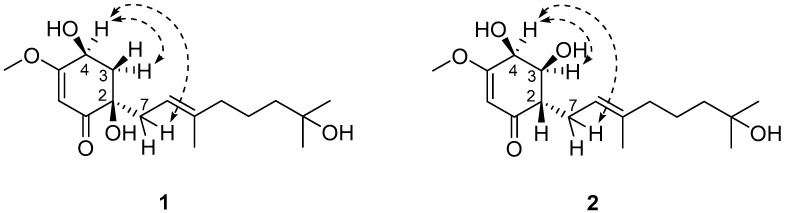
Key ROESY correlations of compounds **1** and **2**.

Sclerotilin A (**1**) was obtained as a brown oil. Its molecular formula was determined to be C_17_H_28_O_5_, indicating four degrees of unsaturation, based on HRESIMS data of the sodium adduct ion at *m*/*z* 355.1829 (calcd. 355.1829 for C_17_H_28_O_5_Na, [M + Na]^+^). The ^1^H NMR spectrum of compound **1** showed signals attributable to two olefinic protons (*δ*_H_ 5.21, 5.36), four methyls (*δ*_H_ 1.21, 1.22, 1.59, 3.81), four methylenes (Table 1), and one methine (*δ*_H_ 4.51). The ^13^C NMR spectrum of compound **1** (Table 1) revealed the presence of 17 carbon signals, comprising 4 methyl carbons (*δ*_C_ 56.8, 29.4, 29.3 and 16.5), 5 methylene carbons (*δ*_C_ 43.2, 40.7, 40.1, 36.9 and 22.4), 3 methine carbons (*δ*_C_ 117.7, 98.7 and 66.2), and 5 quaternary carbons (*δ*_C_ 200.4, 176.3, 139.4, 75.4 and 71.2). The ^1^H-^1^H COSY spectrum revealed three isolated spin-systems: H-3/H-4, H-7/H-8, and H-10/H-11/H-12 (Figure 5). Subsequently, based on the key HMBC correlations from H-6 to C-1, C-2, C-4, C-5, H-3 to C-1, C-5, and H-4 to C-3, C-5, the presence of a cyclohexanone moiety was revealed. Moreover, the key HMBC correlations from H-16 to C-8, C-9, C-10 and from H-14 to C-12, C-13, C-15 revealed the existence of a farnesyl moiety. This farnesyl side chain was located on the cyclohexanone at C-2 based on the key HMBC correlations from H-3 to C-1 and C-7. The structural elucidation revealed that compound **1** is closely related to the known natural product cyclohexenoneterpene D, isolated from the mangrove-associated fungus *Penicillium* sp. N-5 [25]. They possess an identical core skeleton and differ only in the positional arrangement of certain substituents. Compound **1** bears a hydroxyl group at C-13 of the side chain rather than at C-15, supported by HMBC correlations observed from H-14 to C-13 and C-15. Its cyclohexenone ring contains a methoxy substituent at C-5, as evidenced by HMBC correlations from H-17 to C-5 and from H-3 to C-5. Furthermore, two hydroxyl groups are positioned at C-2 and C-4, based on HMBC correlations from H-6 to both C-2 and C-4. Thus, the planar structure of 1 was established as shown in Figure 4. The ROESY correlations of H-4 to H-7 and H-3a *δ*_H_ 2.58 (dd, *J* = 12.8, 5.5 Hz) suggest that H-4 and H-3a *δ*_H_ 2.58 (dd, *J* = 12.8, 5.5 Hz) are in *α*-orientation (Figure 6). Consequently, OH-2, H-3b *δ*_H_ 1.99 (dd, *J* = 12.8, 9.9 Hz), and OH-4 are in *β*-orientation. Thus, the relative configuration of compound **1** is defined as 2R*, 4S* (Figure 6).

Sclerotilin B (**2**) was obtained as a brown oil. The molecular formula of 2 was determined as C_17_H_28_O_5_ by HRESIMS at *m*/*z* 355.1839 [M + Na]^+^ (calcd. for C_17_H_28_O_5_Na, 355.1829), indicating four degrees of unsaturation. Analysis of the ^1^H and ^13^C NMR spectral of **2** revealed structural similarity with **1**. The main difference is that compound **2** lacks one oxygenated quaternary carbon and one methylene group present in **1**, with these being replaced by two methine carbons, indicating that the position of hydroxyl substitution has been changed (Figure 4). Further evidence was provided by ^1^H-^1^H COSY correlations of H-4/H-3/H-2/H-7/H-8 and key HMBC correlations from H-6 to C-2, H-2 to C-4, C-8, and H-3 to C-1, C-5, which collectively confirm the positioning of the two hydroxyl groups on the cyclohexenone ring at C-3 and C-4, respectively (Figure 5). Thus, the analyses of 1D and 2D NMR spectra cumulatively established the structure of compound **2**, as depicted in Figure 4. The ROESY correlations of H-4 to H-7 and H-3 suggest that H-4 and H-3 are in *α*-orientation. Consequently, H-2, OH-3 and OH-4 are in *β*-orientation. Thus, the relative configuration of compound **2** is defined as 2S*, 3S*, 4S* (Figure 6).

### 2.5. Antimicrobial and Cytotoxic Activities

The antimicrobial activity of the isolated compounds was evaluated against five clinically relevant bacterial strains, including *Escherichia coli* ATCC 25922, *Pseudomonas aeruginosa* ATCC 27853, *Staphylococcus aureus* ATCC 6538, methicillin-resistant *Staphylococcus aureus* ATCC 43300, and *Candida albicans* ATCC 10231. The results indicate that compounds **1**, **2**, **3**, **4**, and **7** showed no inhibitory activity against any of the tested pathogens. As detailed in Table 2, compound **5** exhibited weak activity solely against *E. coli* ATCC 25922, with an MIC of 150 µg/mL. In contrast, compound **6** displayed moderate activity against *S. aureus* ATCC 6538 (MIC = 17.36 µg/mL), along with weak inhibition against *E. coli* ATCC 25922 and *P. aeruginosa* ATCC 27853, with higher MIC values of 125 µg/mL and 62.5 µg/mL, respectively.

The cytotoxicity of compounds **1**–**7** was evaluated against five human cancer cell lines: K562 (myeloid leukemia), BEL-7402 (liver cancer), SGC-7901 (gastric cancer), A549 (non-small-cell lung cancer), and HeLa (cervical cancer). As shown in Table 3, only compound **3** exhibited moderate cytotoxic activity across all five cell lines.

Ergosterols have been extensively studied for their anticancer properties. These compounds can exert direct antitumor effects both in vitro and in vivo by mechanisms such as promoting oxidative stress, upregulating the Foxo3/Bim/Fas apoptotic axis, and antagonizing sex hormone receptors, showing activity against laryngeal, breast, prostate, bladder, and melanoma cancers [36,37,38]. In line with these findings, compound **3** (ergosta-5,7,22-trien-3*β*-ol) has been reported to display antiproliferative activity against K562 cells at 20.0 μM, as observed by loss of cell surface roundness and luster under microscopic examination [39]. Furthermore, ergosta-5,7,22-trien-3*β*-ol has demonstrated selective anticolorectal-cancer activity through down-regulation of Bcl-2, up-regulation of Bax and caspase-3, induction of mitochondrial depolarization and ROS accumulation, G2/M phase arrest in HCT116 cells, and molecular docking into the hydrophobic cleft of Bcl-2 to promote apoptosis [40]. Additionally, compound **3** exhibited moderate, dose-dependent growth inhibition against rhabdomyosarcoma (A673), colorectal carcinoma (HCT116), and breast cancer (MCF-7) cells, although it induced detectable apoptosis only at the highest tested concentration of 30 μM [41].

## 3. Materials and Methods

### 3.1. Fungal Identification, Genome Sequencing and BGC Prediction

The strain *P. sclerotiorum* E23Y-1A was originally isolated from the marine sponge *Holoxea* sp. (Quanfu Island, Sansha, China) in March 2019 and was preserved in our laboratory [16]. Wild-type of *P. sclerotiorum* E23Y-1A was cultured on potato dextrose agar (PDA) for 5 days. The spores were scraped from the plate and collected for DNA extraction using the SDS method [42]. The whole genome was sequenced using Illumina NovaSeq PE150 at the Beijing Novogene Bioinformatics Technology Co., Ltd. (Beijing, China). The BGCs were analyzed by the antiSMASH [43].

### 3.2. Preparation of A00667 Knockout Construct

The 1102-bp upstream and 1648-bp downstream sequences of *A00667* were amplified with primer pairs 667up-F/667up-R and 667down-F/667down-R, respectively, and cloned into the EcoRI/ClaI and XhoI/KpnI sites of vector pCT74 to construct a fusion plasmid where the hygromycin resistance cassette (Hyg-R) was flanked by the *A00667* homologous arms. The complete *A00667* knockout construct (upstream-Hyg-R-downstream) was PCR-amplified from the pCT74 plasmid template with primers 667delete-F and 667delete-R. This amplified linear fragment was then used for fungal transformation.

### 3.3. Generation of A00667 Deletion Mutant

Protoplasts of *P. sclerotiorum* were prepared using a two-step enzymatic digestion protocol. Fresh spores were first inoculated into 50 mL of LMM medium and incubated at 28 °C with shaking at 200 rpm for 11 h. The resulting mycelia were harvested by filtration and subjected to the first digestion in an 8 M KCl solution containing 15 mg/mL Yatalase and 10 mg/mL Lysing enzymes at 32 °C for 3 h. After collection by centrifugation at 5000 rpm, the mycelia underwent a second digestion in a fresh 8 M KCl solution containing 15 mg/mL Lywallzyme and 10 mg/mL Driselase under the same conditions (32 °C, 3 h).

The digested mixture was filtered to collect the protoplasts, which were then pelleted by centrifugation at 800× *g* for 10 min at 4 °C. The pellet was washed twice with STC buffer (1.2 M sorbitol, 10 mM CaCl_2_, and 10 mM Tris-HCl, pH 7.5) and finally resuspended in 200 μL of the same buffer. The *A00667* knockout construct was subsequently introduced into the protoplasts using a PEG/CaCl_2_-mediated transformation method as described by Fan et al. [44].

Putative transformants were selected on PDA plates supplemented with 100 μg/mL hygromycin and subjected to PCR confirmation. Correct in-locus recombination was verified using primer pairs 667upcheck/Hyg-R and Hyg-F/667downcheck, while the complete deletion of the *A00667* gene was confirmed using the internal primer pair 667-F/667-R. The verified *A00667* deletion mutants were designated Δ667. Primers used in this study are listed in Table S2.

### 3.4. Fungal Fermentation and Metabolite Isolation

For fermentation, both the wild-type and mutant strain Δ667 were subjected to a 40-day static fermentation at room temperature in eighty 1000 mL flasks, each containing 80 g rice and 120 mL water (autoclaved at 121 °C for 20 min). After fermentation, the cultures were extracted exhaustively with ethyl acetate (EtOAc).

The crude extract was subjected to silica gel column chromatography (CC, 200 mesh) using a gradient elution of petroleum ether/EtOAc (from 9:1 to 1:9, *v*/*v*), followed by CH_2_Cl_2_/MeOH (9:1 *v*/*v*), yielding twenty fractions (E1–E20) from the wild type E23Y-1A and eighteen fractions (C1–C18) from the mutant strain Δ667. Compound **5** (4.0 mg) was obtained from fraction E4 using silica gel CC eluted with petroleum ether/EtOAc (8:1, *v*/*v*). Fraction E17 was separated by silica gel CC eluted with petroleum ether/EtOAc (5:1, *v*/*v*) to yield **7** (5.0 mg) and then eluted with CH_2_Cl_2_/MeOH (40:1, *v*/*v*), followed with semi-preparative HPLC (MeOH/H_2_O, 70:30, *v*/*v*) to afford **6** (60.0 mg, *t*_R_ = 7.1 min).

Fraction C2 was treated with 100% methanol and centrifugated at 12,000 rpm to give a chloroform-soluble precipitate, compound **3** (3.0 mg). Fraction C10 was purified by gel silica CC with a stepwise CH_2_Cl_2_/MeOH elution (80:1 to 20:1, *v*/*v*) to give **4** (1.5 mg) and nine subfractions (C10A–C10I). Subfraction C10E was purified by silica gel CC (CH_2_Cl_2_/MeOH, 40:1, *v*/*v*) and subsequent semi-preparative HPLC using (MeOH/H_2_O, 70:30, *v*/*v*) to afford compound **2** (1.6 mg, *t*_R_ = 6.9 min). Similarly, subfraction C10G was purified by silica gel CC (CH_2_Cl_2_/MeOH, 80:1, *v*/*v*) followed by semi-preparative HPLC (MeOH/H_2_O, 70:30, *v*/*v*) to give **1** (2.9 mg, *t*_R_ = 7.7 min).

Compound **1**: brown oil; [*α*${]}_{D}^{25}$: −13.0 (*c* 0.1, MeOH); UV (MeOH) *λ*_max_ (log*ε*) 259 (2.71) nm; HRESIMS *m*/*z*: 355.1829 [M + Na]^+^ (calcd. for 355.1829, C_17_H_28_O_5_Na); ^1^H NMR (600 MHz) and ^13^C NMR (125 MHz) data in CDCl_3_; see Table 1.

Compound **2**: brown oil; [*α*${]}_{D}^{25}$: +31.0 (*c* 0.1, MeOH); UV (MeOH) *λ*_max_ (logε) 252 (2.58) nm; HRESIMS *m*/*z*: 355.1839 [M + Na]^+^ (calcd. for 355.1829, C_17_H_28_O_5_Na); ^1^H NMR (600 MHz) and ^13^C NMR (125 MHz) data in CDCl_3_; see Table 1.

### 3.5. HPLC Analysis on Crude Extracts of the Wild Strain E23Y-1A and the Mutant Strain Δ667

Crude extracts of both E23Y-1A and Δ667 were prepared at a concentration of 1 mg/mL for HPLC analysis using a gradient elution starting with MeOH/H_2_O (5:95, *v*/*v*) for 5 min, followed by a linear increase to 100% MeOH over 45 min, and then an isocratic hold at 100% MeOH for 10 min. the flow rate was 0.5 mL/min with UV detection at 365 nm and 254 nm.

### 3.6. General Experimental Procedures

^1^H and ^13^C NMR data were acquired using Bruker Avance III 400 and DRX 600 spectrometers (Bruker Biospin AG, Ettlingen, Germany). All chemical shifts are referenced to tetramethylsilane (TMS) and reported in parts per million (ppm), while coupling constants are presented in Hertz (Hz). HRESIMS analyses were performed on an Agilent G6520 Q-TOF mass spectrometer (Agilent, Santa Clara, CA, USA). Column chromatography was performed using silica gel (200–300 and 300–400 mesh; Qingdao Haiyang Chemical Group Co., Ltd., Qingdao, China) and Sephadex LH-20 (Merck, Darmstadt, Germany). Optical rotations were measured with a Perkin–Elmer 241MC polarimeter (PerkinElmer, Fremont, CA, USA). ECD and UV data were acquired using a Jasco J-810 spectropolarimeter (JASCO, Tokyo, Japan). Infrared (IR) spectra were recorded on a Nicolet 380 FT-IR spectrometer (Thermo, Waltham, MA, USA) using samples prepared as KBr pellets. HPLC separation was conducted using an Agilent 1260 series system employing a DAD G1315D detector and an Eclipse XDB-C18 column (5 μm, 9.4 × 250 mm) (Agilent, Santa Clara, CA, USA).

### 3.7. Antimicrobial Activity Assay

The minimum inhibitory concentrations (MICs) of the isolated compounds against *Escherichia coli* ATCC 25922, *Pseudomonas aeruginosa* ATCC 27853, *Staphylococcus aureus* ATCC 6538, methicillin-resistant *Staphylococcus aureus* ATCC 43300, and *Candida albicans* ATCC 10231 were determined using a 96-well microtiter plate broth microdilution assay. Each test compound was dissolved in dimethyl sulfoxide (DMSO), and 5 µL of the resulting solution was transferred into nutrient broth to prepare a series of two-fold serial dilutions. Subsequently, 100 µL of each compound dilution was combined with 100 µL of a bacterial suspension which was adjusted to approximately 1.0 × 10^5^ CFU/mL in each well. Levofloxacin or vancomycin were employed as positive controls, while DMSO served as the negative control. Following incubation at 25 °C for 24 h, the MIC was determined visually as the lowest concentration at which no turbidity was observed. All experiments were conducted in triplicate to ensure reproducibility.

### 3.8. Cytotoxic Detection

All compounds were evaluated for cytotoxic activity against five human tumor cell lines: K562 (myeloid leukemia), BEL-7402 (liver cancer), SGC-7901 (gastric cancer), A549 (non-small-cell lung cancer), and HeLa (cervical cancer). These cell lines were obtained from the Cell Bank of the Type Culture Collection at the Shanghai Institute of Cell Biology, Chinese Academy of Sciences. Cytotoxicity was assessed using a modified MTT assay as described by Zeng et al. [16]. Absorbance measurements were performed at 570 nm using a Multiskan FC photometric microplate reader (Thermo Fisher Scientific). Cisplatin served as the positive control.

## 4. Conclusions

In this study, we successfully disrupted the azaphilone biosynthetic pathway in *P. sclerotiorum* E23Y-1A by deleting the regulatory gene *A00667*, leading to the suppression of azaphilone production and the emergence of new metabolites. From the mutant strain, two novel meroterpenoids sclerotilins A and B (**1** and **2**) along with two known steroids were isolated and structurally characterized. The antimicrobial screening showed that most compounds were inactive; however, compound **5** exhibited weak activity against *E. coli* ATCC 25922, and compound **6** showed moderate activity against *S. aureus* ATCC 6538 along with weak effects against *E. coli* and *P. aeruginosa*. In cytotoxicity testing, only compound **3** exhibited moderate activity against all five tested human cancer cell lines—K562, BEL-7402, SGC-7901, and A549. The research confirms *A00667* as a key regulator of azaphilone biosynthesis and demonstrates that metabolic shunting through genetic manipulation can effectively redirect fungal metabolism to yield novel natural products.

## Figures and Tables

**Table 1:** ^1^H NMR (600 MHz) and ^13^C NMR (125 MHz) spectral data of compounds **1** and **2** in CDCl_3_.

	1		2	
NO.	*δ*_C_	*δ*_H_ *(J* in Hz)	*δ*_C_	*δ*_H_ (*J* in Hz)
1	200.4, C		198.1, C	
2	75.4, C		50.0, CH	2.71, dt (7.4, 5.5)
3	40.7, CH_2_	2.58, dd (12.8, 5.5) 1.99, dd (12.8, 9.9)	71.0, CH	4.10, dd (6.9, 3.7)
4	66.2, CH	4.51, dd (10.0, 5.5)	68.1, CH	4.46, d (3.8)
5	176.3, C		171.7, C	
6	98.7, CH	5.36, s	102.4, CH	5.38, s
7	36.9, CH_2_	2.34, dd (14.7, 8.4) 2.23, dd (14.7, 6.3)	26.1, CH_2_	2.39, m; 2.46, m
8	117.7, CH	5.21, dd (8.4, 6.3)	121.3, CH	5.18, t (7.4)
9	139.4, C		138.1, C	
10	40.1, CH_2_	2.03, t (6.9)	40.1, CH_2_	1.99, t (7.0)
11	22.4, CH_2_	1.46, m	22.5, CH_2_	1.44, m
12	43.2, CH_2_	1.43, m	43.4, CH_2_	1.41, m
13	71.2, C		71.2, C	
14	29.4, CH_3_	1.21, s	29.5, CH_3_	1.21, s
15	29.3, CH_3_	1.22, s	29.4, CH_3_	1.21, s
16	16.5, CH_3_	1.59, s	16.3, CH_3_	1.65, s
17	56.8, CH_3_	3.81, s	56.5, CH_3_	3.77, s

**Table 2:** Minimum inhibitory concentration (MIC) values of compounds **5**, **6** and positive control antibiotics against selected bacterial strains.

Strain	MIC (µg/mL)			
	5	6	Levofloxacin ^a^	Vancomycin ^b^
*E. coli* ATCC 25922	150	125	0.195	NT
*P. aeruginosa* ATCC 27853	ND	62.5	0.390	NT
*S. aureus* ATCC 6538	ND	17.36	NT	3.47

^a^ positive antibiotic against *E.coli* and *P. aeruginosa.* ^b^ positive antibiotic against *S. aureus.*

**Table 3:** Cytotoxic activity of compound **3**.

Compound	IC_50_ ± SD (μM) ^a^				
	K562	BEL-7402	SGC-7901	A549	Hela
3	26.18 ± 0.35	54.91 ± 0.28	34.83 ± 0.10	42.87 ± 0.40	47.79 ± 0.43
Cisplatin ^b^	3.08 ± 0.05	4.02 ± 0.06	4.11 ± 0.02	1.93 ± 0.02	11.29 ± 0.15

^a^ Values represent means *±* SD based on three parallel experiments. ^b^ positive control.

## Data Availability

The authors confirm that the data supporting the findings of this study are available within the article and its Supplementary Materials.

## Supplementary Materials

The following supporting information can be downloaded at https://www.mdpi.com/article/10.3390/md24030095/s1: Table S1: Proteins predicted in azaphilone BGC of *P. sclerotiorum* E23Y-1A; Table S2: Primers used in study; Table S3: Physical and spectroscopic characterization data for compounds **3**–**7**; Figures S1–S9: 1D NMR (^1^H NMR, ^13^C NMR), 2D NMR (DEPT, HSQC, HMBC, ^1^H-^1^H COSY, ROESY), HRESIMS, and UV spectra of compound **1**; Figures S10–S18: 1D NMR (^1^H NMR, ^13^C NMR), 2D NMR (DEPT, HSQC, HMBC, ^1^H-^1^H COSY, ROESY), HRESIMS, and UV spectra of compound **2**; Figures S19–S23: 1D NMR (^1^H NMR, ^13^C NMR), 2D NMR (DEPT, HSQC), and HRESIMS spectra of compound **3**; Figures S24–S28: 1D NMR (^1^H NMR, ^13^C NMR), 2D NMR (DEPT, HSQC), and HRESIMS spectra of compound **4**; Figures S29–S33: 1D NMR (^1^H NMR, ^13^C NMR), 2D NMR (DEPT, HSQC), and HRESIMS spectra of compound **5**; Figures S34–S38: 1D NMR (^1^H NMR, ^13^C NMR), 2D NMR (DEPT, HSQC), and HRESIMS spectra of compound **6**; Figures S39–S43: 1D NMR (^1^H NMR, ^13^C NMR), 2D NMR (DEPT, HSQC), and HRESIMS spectra of compound **7**.
