# Supplementary material for: Generation of Novel Natural Products by Disrupting Azaphilone Synthesis in Penicillum sclerotiorum E23Y-1A
Source: Mar Drugs. 2026 Feb 27;24(3):95. doi: 10.3390/md24030095 (PMC13028492; doi:10.3390/md24030095)
Supplement: Supplementary file 1 [file marinedrugs-24-00095-s001.zip › marinedrugs-4144221-supplementary.pdf]

Supporting information

Generation of Novel Natural Products by Disrupting Azaphilone Synthesis in *Penicillum sclerotiorum* E23Y-1A

Wenjun Chang [1,†], Yanhua Yang [2,†], Ruijun Duan [1], Heye Qin [1], Shiwen Chen [1], and Yanbo Zeng [1,3,*]

[1] Hainan Provincial Key Laboratory for Functional Components Research and Utilization of Marine Bio-resources, Institute of Tropical Bioscience and Biotechnology, Chinese Academy of Tropical Agricultural Sciences & Key Laboratory for Biology and Genetic Resources of Tropical Crops of Hainan Province, Hainan Institute for Tropical Agricultural Resources, Haikou 571101, China

[2] School of Pharmacy and Bioengineering, Chongqing University of Technology, Chongqing 400054, China

[3] Hainan Key Laboratory for Biosafety Monitoring and Molecular Breeding in Off-Season Reproduction Regions, Key Laboratory of Biology and Genetic Resources of Tropical Crops, Sanya Research Institute of Chinese Academy of Tropical Agricultural Sciences, Sanya 572025, China

* Correspondence: zengyanbo@catasitbb.cn

† These authors contributed equally to this work.

Table S1. Proteins predicted in the azaphilone BGC of *P. sclerotiorum* E23Y-1A
Table S2. Primers used in this study
Table S3. Physical and spectroscopic characterization data for compounds 3-7
Figure S1. ^{1}H NMR (600 MHz, $CDCl_3$) spectrum of compound 1
Figure S2. ^{13}C NMR (125 MHz, $CDCl_3$) spectrum of compound 1
Figure S3. DEPT spectrum of compound 1
Figure S4. HSQC spectrum of compound 1
Figure S5. HMBC spectrum of compound 1
Figure S6. ^{1}H-^{1}H COSY spectrum of compound 1
Figure S7. ROESY spectrum of compound 1
Figure S8. HRESIMS spectrum of compound 1
Figure S9. UV spectrum of compound 1
Figure S10. ^{1}H NMR (600 MHz, $CDCl_3$) spectrum of compound 2
Figure S11. ^{13}C NMR (125 MHz, $CDCl_3$) spectrum of compound 2
Figure S12. DEPT spectrum of compound 2
Figure S13. HSQC spectrum of compound 2
Figure S14. HMBC spectrum of compound 2
Figure S15. ^{1}H-^{1}H COSY spectrum of compound 2
Figure S16. ROESY spectrum of compound 2
Figure S17. HRESIMS spectrum of compound 2
Figure S18. UV spectrum of compound 2
Figure S19. ^{1}H NMR (600 MHz, $CDCl_3$) spectrum of compound 3
Figure S20. ^{13}C NMR (125 MHz, $CDCl_3$) spectrum of compound 3
Figure S21. DEPT spectrum of compound 3
Figure S22. HSQC spectrum of compound 3
Figure S23. HRESIMS spectrum of compound 3
Figure S24. ^{1}H NMR (600 MHz, $CDCl_3$) spectrum of compound 4
Figure S25. ^{13}C NMR (125 MHz, $CDCl_3$) spectrum of compound 4
Figure S26. DEPT spectrum of compound 4
Figure S27. HSQC spectrum of compound 4
Figure S28. HRESIMS spectrum of compound 4
Figure S29. ^{1}H NMR (600 MHz, $CDCl_3$) spectrum of compound 5
Figure S30. ^{13}C NMR (125 MHz, $CDCl_3$) spectrum of compound 5
Figure S31. DEPT spectrum of compound 5
Figure S32. HSQC spectrum of compound 5
Figure S33. HRESIMS spectrum of compound 5
Figure S34. ^{1}H NMR (600 MHz, MeOD) spectrum of compound 6
Figure S35. ^{13}C NMR (125 MHz, MeOD) spectrum of compound 6
Figure S36. DEPT spectrum of compound 6
Figure S37. HSQC spectrum of compound 6
Figure S38. HRESIMS spectrum of compound 6
Figure S39. ^{1}H NMR (600 MHz, $CDCl_3$) spectrum of compound 7
Figure S40. ^{13}C NMR (125 MHz, $CDCl_3$) spectrum of compound 7
Figure S41. DEPT spectrum of compound 7

Figure S42. HSQC spectrum of compound 7
Figure S43. HRESIMS spectrum of compound 7

Table S1. Proteins predicted in the azaphilone BGC of *P. sclerotiorum* E23Y-1A.

Protein	Size (AA)	Putative function
AA650	274	SDR family NAD(P)-dependent oxidoreductase
AA651	593	trichothecene efflux pump
AA652	801	fungal-specific transcription factor domain-containing protein
AA653	485	aldehyde dehydrogenase
AA654	341	enoyl reductase-like protein
AA655	471	Dyp-type peroxidase
AA656	672	galactose oxidase
AA657	320	hypothetical protein
AA660	411	hypothetical protein, integral membrane protein
AA661	490	FAD-linked oxidoreductase
AA662	2743	NRPKS (**PX533544**)
AA663	1005	hypothetical protein
AA664	449	acyltransferase (**PX533545**)
AA665	632	efflux pump (**PX533546**)
AA666	387	alcohol dehydrogenase (**PX533547**)
AA667	590	putative Zn(II)$_2$Cys$_6$ transcription factor (**PX533548**)
AA668	458	FAD-dependent monooxygenase (afoD, **PX533549**)
AA669	561	Trp halogenase (**PX533550**)
AA671	593	hypothetical protein
AA672	2588	HRPKS (**PX533551**)
AA673	883	phytoene desaturase
AA674	552	phytoene desaturase
AA675	318	hypothetical protein
AA676	720	hypothetical protein
AA677	126	thioredoxin-like protein
AA678	311	ribosomal protein S5 domain 2-type fold subgroup
AA679	413	4-diphosphocytidyl-2C-methyl-D-erythritol synthase
AA680	544	2-C-methyl-D-erythritol 2,4-cyclodiphosphate synthase
AA682	556	major facilitator super transporter

PX533544-PX533551 are the GenBank accession numbers for the proteins.

Table S2. Primers used in this study.

primer	sequence (5′-3′)	application
667up-F	AGgaattcGCCTCACTTATCGGCTCGTT	667 upstream (EcoRI)
667up-R	ATatcgatGCTGATGTTACTGCTGGGGTC	667 upstream (ClaI)
667down-F	TGctcgagACCTAACTGGGCGAACGG	667 downstream (XhoI)
667down-R	AAggtaccACCACGGGAATCCAAACG	667 downstream (KpnI)
667delete-F	GCCTCACTTATCGGCTCGTT	667 knockout construct

667delete-R	ACCACGGGAATCCAAACG	667 knockout construct
667upcheck	CCTCGCCTACTAAGCGTCAGA	diagnosis
Hyg-R	CAACCACGGCCTCCAGAA	diagnosis
Hyg-F	CCTATTCTACCCAAGCATCCAA	diagnosis
667downcheck	GGAGGAGGAAAGGTGATGTG	diagnosis
667-F	ACGCAGACACCGTTATTACAGA	diagnosis
667-R	TCCACCGTTCGCCAAGAG	diagnosis

Table S3. Physical and spectroscopic characterization data for compounds **3-7**.

**Compound 3**: white powder, HHRESIMS *m/z* 419.3287 $[M+Na]^+$ (calcd. for 419.3284, $C_{28}H_{44}NaO$); $[\alpha]25$ D= -55(c 0.1, $CHCl_3$). ^{13}C NMR (126 MHz, $CDCl_3$) δ 141.5 (C-8), 139.9 (C-9), 135.7 (C-22), 132.1 (C-23), 119.7 (C-6), 116.4 (C-7), 70.6 (C-3), 55.9 (C-17), 54.7 (C-14), 46.4 (C-9), 42.9 (C-13), 42.9 (C-24), 40.9 (C-4), 40.6 (C-20), 39.2 (C-12), 38.5 (C-1), 37.2 (C-10), 33.2 (C-25), 32.1 (C-2), 28.4 (C-16), 23.1 (C-15), 21.2 (C-21), 21.2 (C-11), 20.1 (C-27), 19.7 (C-26), 17.7 (C-28), 16.4 (C-19), 12.2 (C-18);^1H NMR (500 MHz, $CDCl_3$) δ 5.57 (1H, dd, J = 5.7, 2.6 Hz, H-6), 5.38 (1H, dt, J = 5.6, 2.8 Hz, H-7), 5.22 (1H, dd, *J* = 15.3, 7.1 Hz, H-23), 5.18 (1H, d, *J* = 7.6 Hz, H-22), 1.03 (3H, d, J = 6.6 Hz, H-21), 0.94 (3H, s, H-19), 0.91 (3H, d, J = 6.8 Hz, H-28), 0.83 (3H, d, J = 7.2 Hz, H-26), 0.84 (3H, d, J = 7.2 Hz, H-27), 0.63 (3H, s, H-18).
**Compound 4**: white powder, HHRESIMS *m/z* 453.3335 $[M+Na]^+$ (calcd. for 453.3339, $C_{28}H_{46}NaO_3$); $[\alpha]25$ D= -11(c 0.1, CH_3OH). ^{13}C NMR (126 MHz, $CDCl_3$)δ144.44(C-8), 135.78(C-22), 132.58(C-23), 117.94(C-7), 76.37(C-5), 74.07(C-6), 68.14(C-3), 56.38(C-17), 55.16(C-14), 44.17(C-13), 43.88(C-9), 43.22(C-24), 40.82(C-20), 39.86(C-4), 39.62(C-12), 37.54(C-10), 33.48(C-1), 33.37(C-25), 31.26(C-2), 28.32(C-16), 23.29(C-15), 22.45(C-11), 21.52(C-21), 20.36(C-26), 20.05(C-27), 19.26(C-19), 17.99(C-28), 12.74(C-18).^1H NMR (500 MHz, $CDCl_3$) δ 5.35 (2H,m, H-7), 5.22 (1H,dd, J = 15.3, 7.4 Hz, H-23), 5.16 (1H,dd, J = 15.3, 8.2 Hz, H-22), 4.08 (3H, tt, J = 10.9, 4.8 Hz, H-3), 3.62 (1H,d, J = 5.3 Hz, H-6), 2.14(1H,m,H-12a), 2.07(1H,m,H-4a), 2.04(1H,,m,H-20), 1.92 -1.95(1H,m, H-9), 1.90(1H,m,H-14), 1.85-1.87(1H,m,H-2a), 1.84(1H,, m,H-24), 1.78(1H,m,H-4b), 1.74(1H,,m,H-16b), 1.59-1.62(2H,m,H-1), 1.50-1.59(2H,m,H-11), 1.48(1H,,m,H-25), 1.45-1.48(2H,m,H-15), 1.42-1.45(1H,m,H-2b), 1.32(1H,m,H-12b), 1.30(1H,, m,H-16a), 1.08 (1H,s, H-19), 1.02 (1H,d, J = 6.6 Hz, H-21), 0.91 (1H,d, J = 6.8 Hz, H-28), 0.84 (1H,d, J = 6.7 Hz, H-26), 0.82 (1H,d, J = 6.8 Hz,H-27), 0.60 (1H,s, H-18).
**Compound 5**: white powder, HRESIMS *m/z* 451.3188 $[M+Na]^+$ (calcd. for 451.3183, $C_{28}H_{44}NaO_3$); $[\alpha]25$ D=-69.9 (c 0.1, CH_3OH). ^1H NMR (500 MHz, $CDCl_3$) δ 6.50 (1H,d, *J* = 8.5 Hz,H-7), 6.24 (1H,d, *J* = 8.5 Hz,H-6), 5.22 (1H,dd, *J* = 15.2, 7.6 Hz, H-23), 5.13 (1H,dd, *J* = 15.3, 8.3 Hz,H-22), 4.03~3.92 (1H,m,H-3), 0.99 (3H,d, *J* = 6.6 Hz, H-21), 0.90 (3H,d, *J* = 6.9 Hz, H-28), 0.88 (3H,s,H-19), 0.82 (3H,d, *J* = 6.8 Hz, H-26),0.83 (3H,d, *J* = 6.8 Hz, H-27), 0.81 (3H,s,H-18); ^{13}C NMR (126 MHz, $CDCl_3$) δ 135.5 (C-6), 135.3 (C-22), 132.4 (C-23), 130.9 (C-7), 82.3 (C-5), 79.6 (C-8), 66.6 (C-3), 56.3 (C-17), 51.8 (C-14), 51.2 (C-9), 44.7 (C-13), 42.9 (C-24), 39.9 (C-20), 39.5 (C-12), 37.1 (C-10), 37.1 (C-4), 34.8 (C-1), 33.2 (C-25), 30.3 (C-2), 28.8 (C-16), 23.5 (C-11), 21.0 (C-21), 20.8 (C-15), 20.1 (C-27), 19.8 (C-26), 18.3 (C-19), 17.7 (C-28), 13.0 (C-18).
**Compound 6**: yellow oil, HHRESIMS *m/z* 409.1398 $[M+Na]^+$ (calcd. for 409.1388, $C_{19}H_{27}ClNaO_6$); $[\alpha]25$ D=-114 (c 0.1, CH_3OH). ^1H NMR (500 MHz, MeOD) δ 6.73 (1H,d, *J* = 15.6 Hz, H-10), 6.24 (1H,d, *J* = 15.7 Hz, H-9), 6.06 (1H,s,H-4), 4.76 (1H,dd, *J* = 10.8, 5.2 Hz, H-1a), 3.79

(1H,dd, *J* = 13.7, 10.9 Hz,H-1b), 3.40 (1H,d, *J* = 8.3 Hz, H-8), 3.38 (1H,s, H-12), 3.13 (1H,m, H-8a), 1.69(1H,m,H-13), 1.6 (1H,m,H-14b),1.39(1H,m,H-14a), 1.29 (3H,s,H-16), 0.87 (3H,t, *J* = 7.4 Hz,H-15), 0.83 (3H,d, *J* = 6.7 Hz, H-17);^{13}C NMR (126 MHz, MeOD) δ 191.5 (C-6), 164.1 (C-3), 148.1 (C-4a), 145.2 (C-10), 122.6 (C-9), 120.0 (C-5), 102.0 (C-4), 80.9 (C-12), 76.8 (C-11), 76.6 (C-7), 74.5 (C-8), 70.1 (C-1), 38.5 (C-8a), 36.4 (C-13), 30.0 (C-14), 26.4 (C-16), 20.1 (C-18), 14.3 (C-17), 12.2 (C-15).
**Compound 7:** yellow oil, HHRESIMS *m/z* 409.1385 $[M+Na]^+$ (calcd. for 409.1388, $C_{19}H_{27}ClNaO_{6}$,); [α]25 D= +52(c 0.1, CH_3OH). ^{1}H NMR (500 MHz, $CDCl_3$) δ 6.56 (1H d, *J* = 15.5 Hz, H-10), 6.29 (1H, d, *J* = 15.5 Hz, H-9), 6.10 (1H, s, H-4), 4.50 (1H, dd, *J* = 11.2, 4.9 Hz, H-1a), 4.25 (1H, dd, *J* = 13.2, 11.1 Hz, H-1b), 4.12 (1H, d, *J* = 3.0 Hz, H-8), 3.51 (1H, d, *J* = 2.3 Hz, H-12), 3.06 (1H, m, H-8a), 1.71 (1H, m, H-13), 1.37 (3H, s, H-16), 1.32 (3H, s, H-17), 0.97 (3H, d, *J* = 6.8 Hz, H-18), 0.92 (3H, t, *J* = 7.4 Hz, H-15); ^{13}C NMR (126 MHz, $CDCl_3$) δ 193.2 (C-6), 161.3 (C-2), 145.2 (C-10), 144.3 (C-10), 122.8 (C-9), 116.7 (C-5), 102.9 (C-4), 78.3 (C-12), 77.6 (C-11), 75.9 (C-8), 73.8 (C-7), 68.4 (C-1), 36.9 (C-8a), 35.5 (C-13), 28.8 (C-14), 23.7 (C-17), 23.5 (C-16), 13.5 (C-18), 12.0 (C-15).

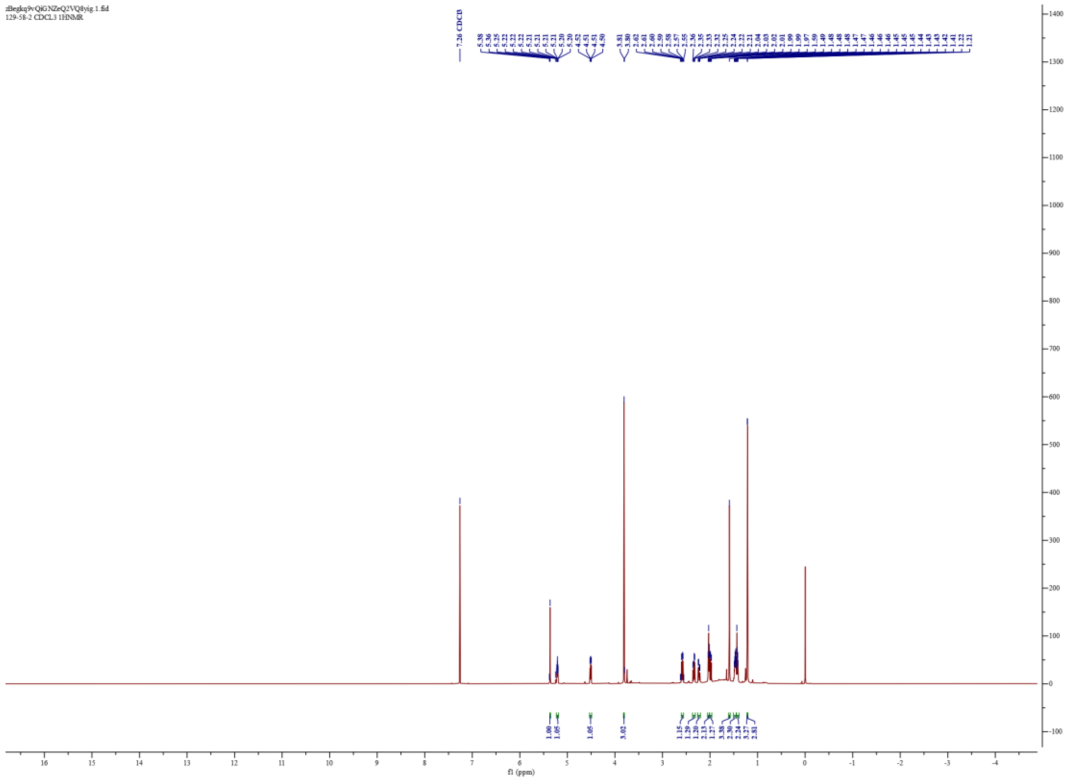

Figure S1. ^{1}H NMR (600 MHz, $CDCl_3$) spectrum of compound 1.

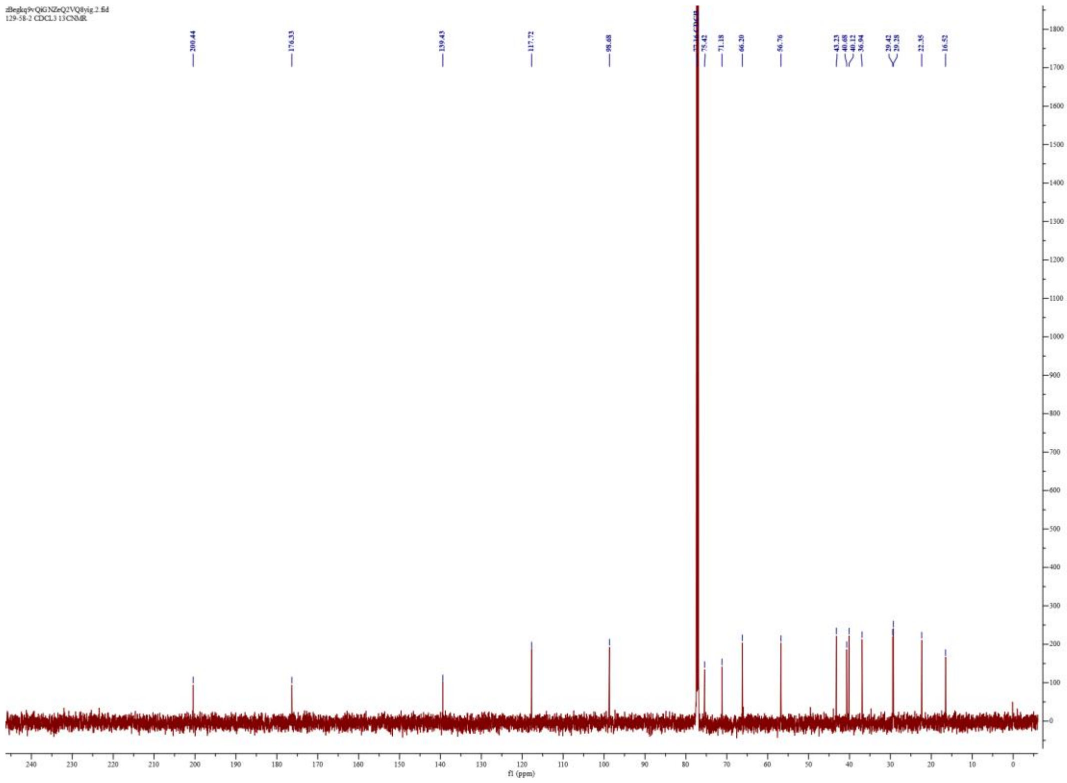

Figure S2. ^{13}**C NMR (125 MHz, CDCl$_3$) spectrum of compound 1.**

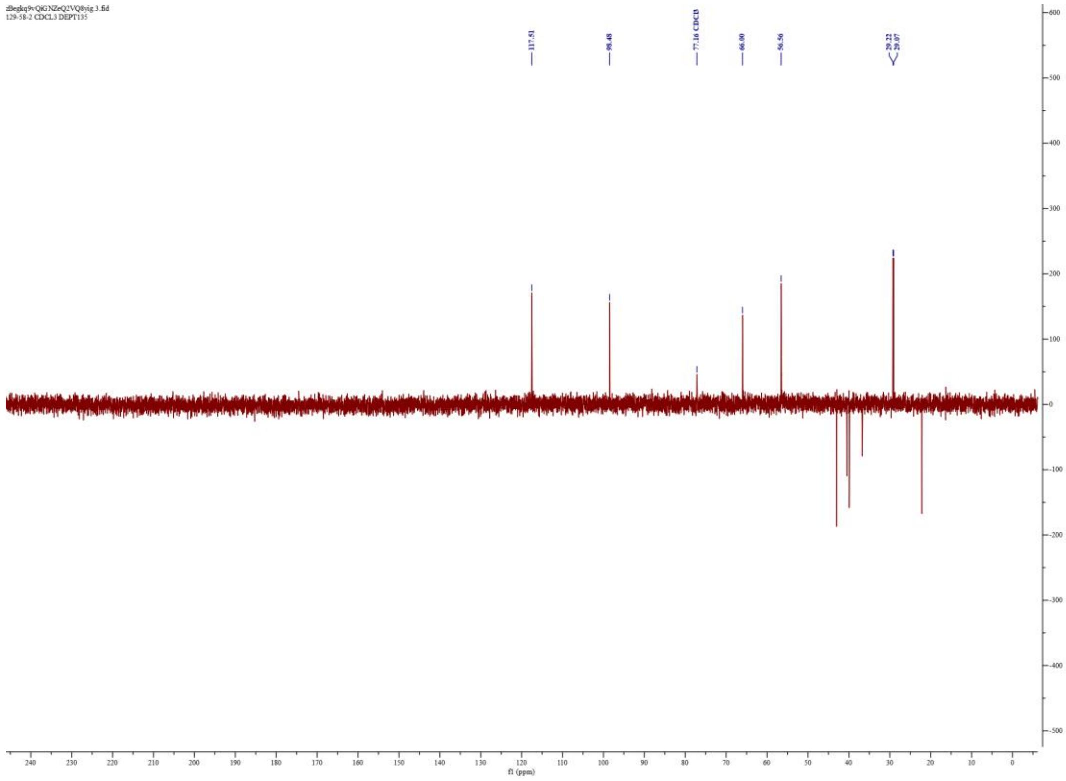

Figure S3. DEPT spectrum of compound 1.

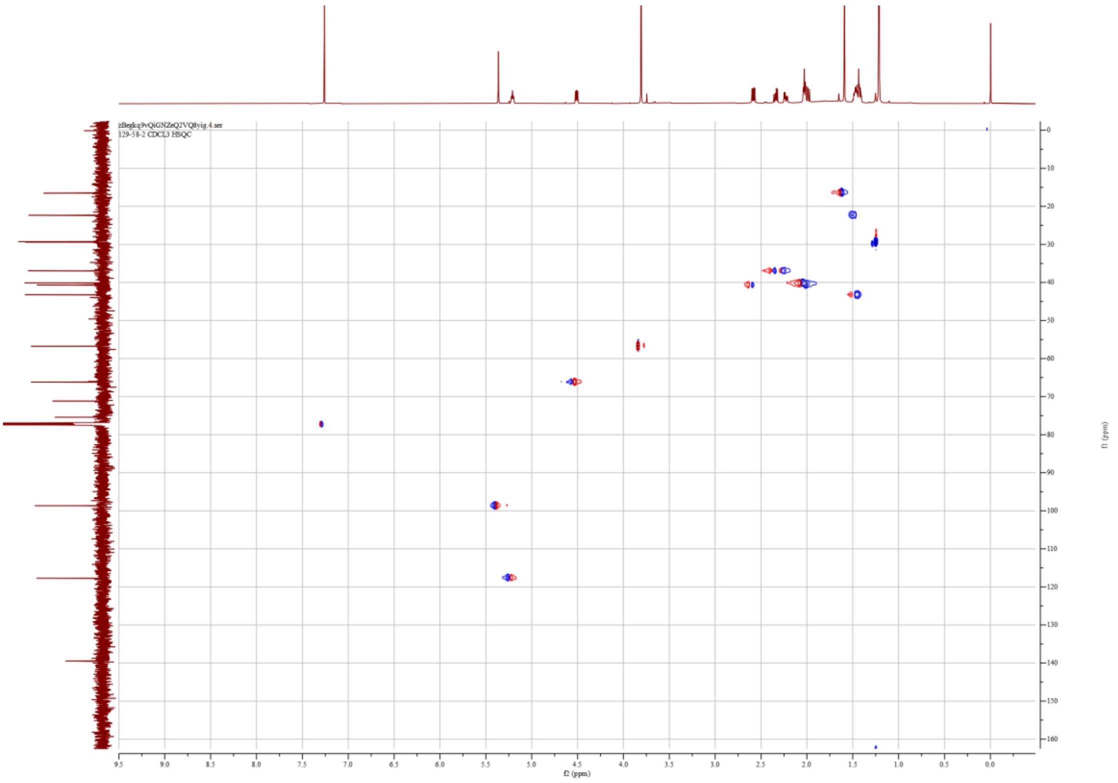

Figure S4. HSQC spectrum of compound 1.

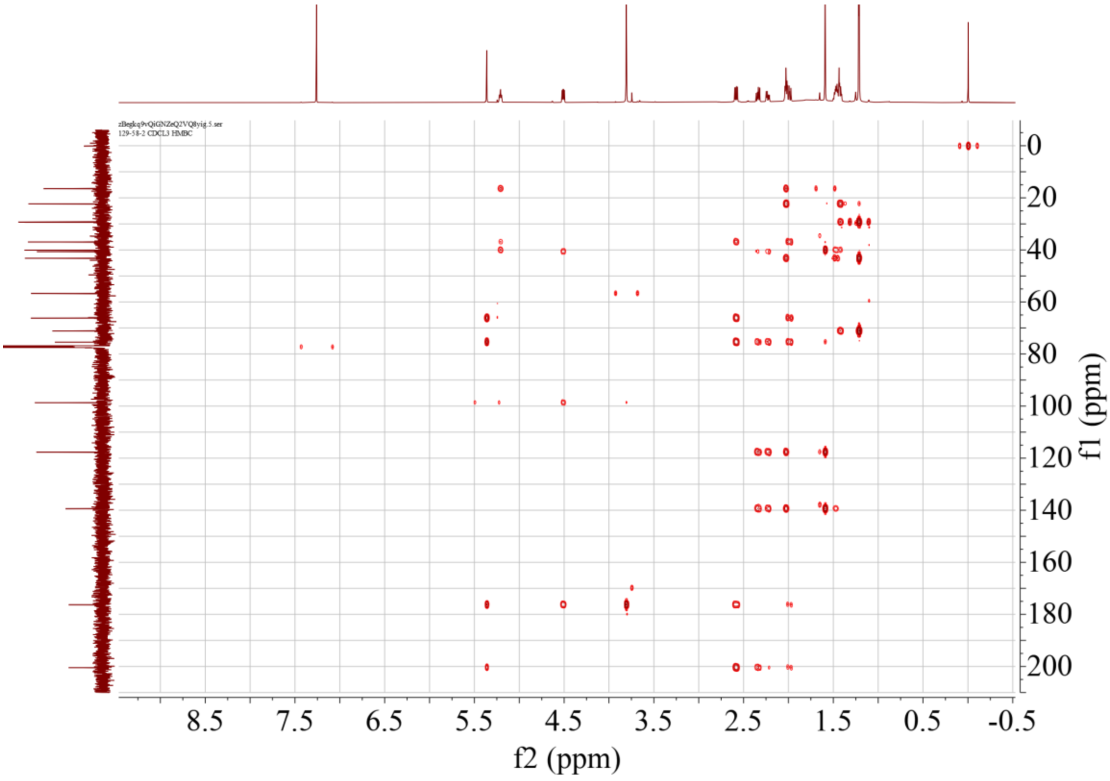

Figure 5S. HMBC spectrum of compound 1.

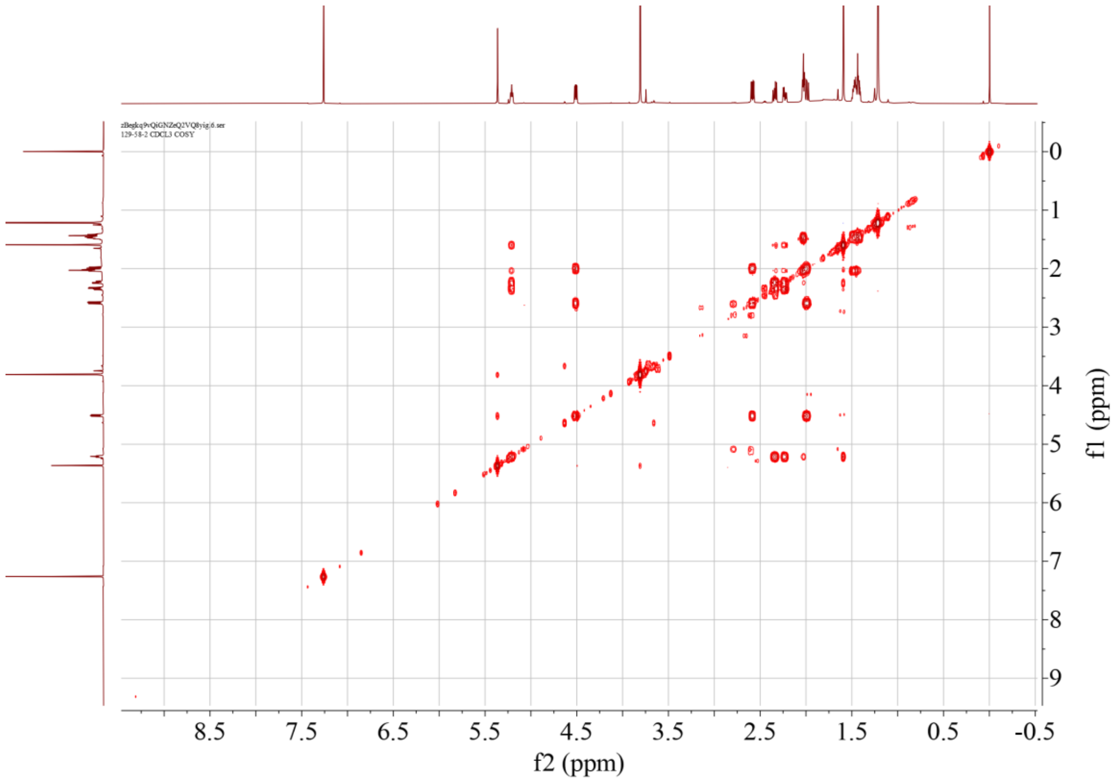

Figure 6S. ^{1}H-^{1}H COSY spectrum of compound 1.

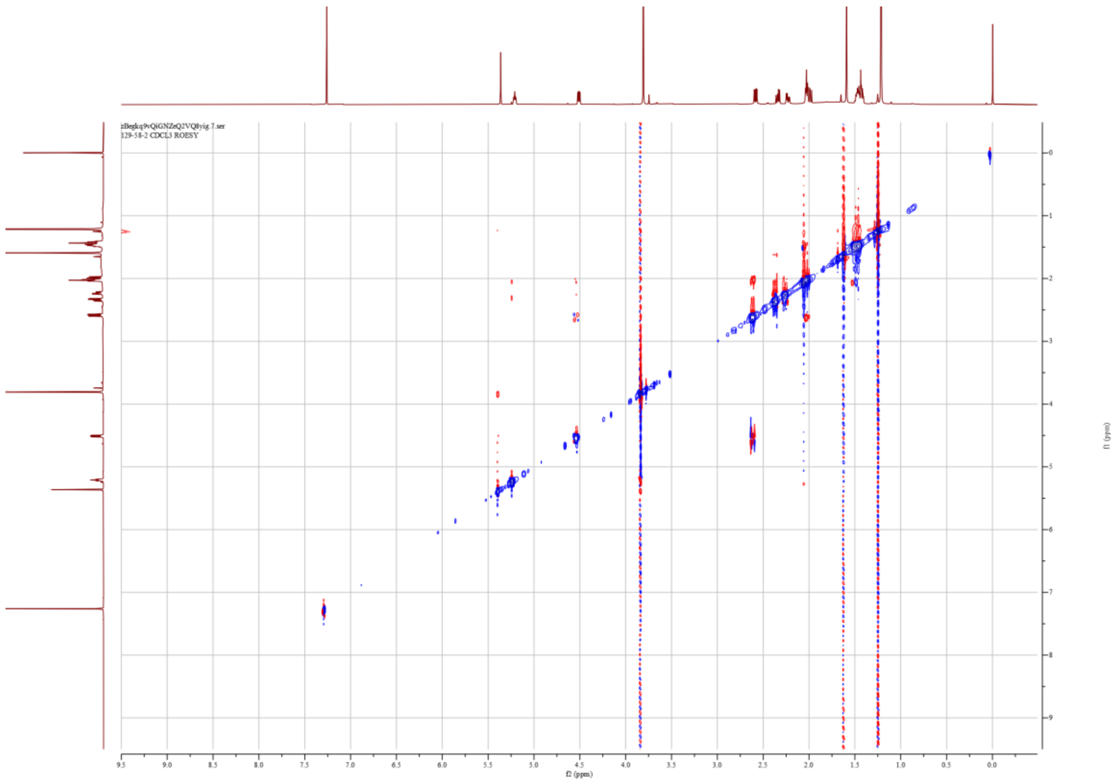

Figure 7S. ROESY spectrum of compound 1.

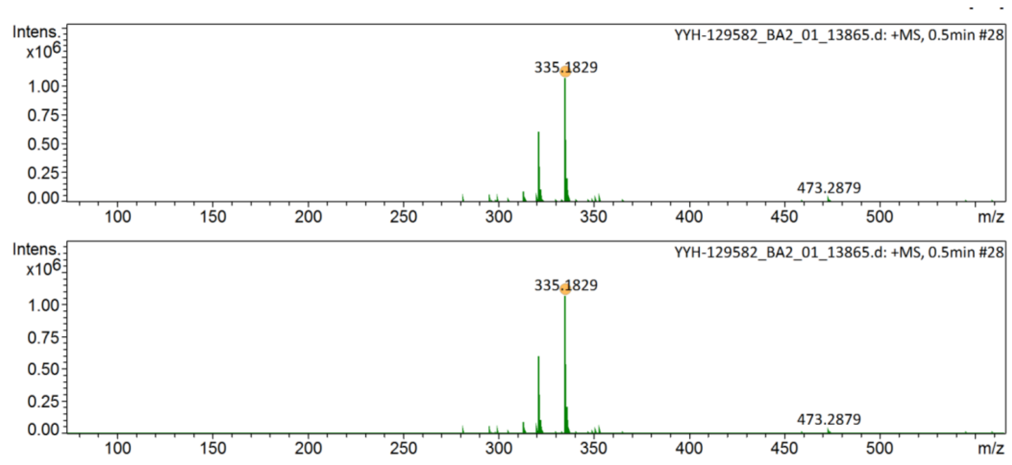

Meas. m/z	#	Ion Formula	m/z	err [ppm]	mSigma	# mSigma	Score	rdb	e⁻ Conf	N-Rule	z	Adduct
335.1829	1	C17H28NaO5	335.1829	-0.1	3.5	1	100.00	4.0	even	ok	1+	M+Na

Figure 8S. HRESIMS spectrum of compound 1.

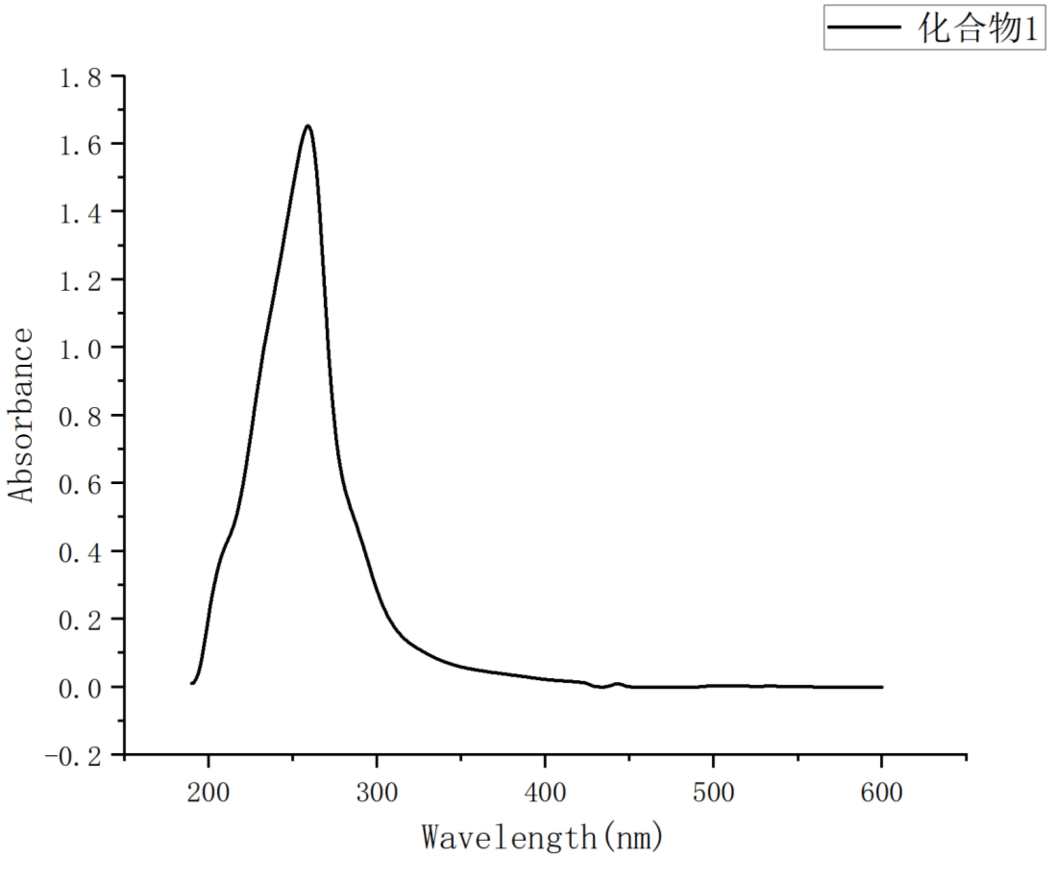

Figure 9S. UV spectrum of compound 1.

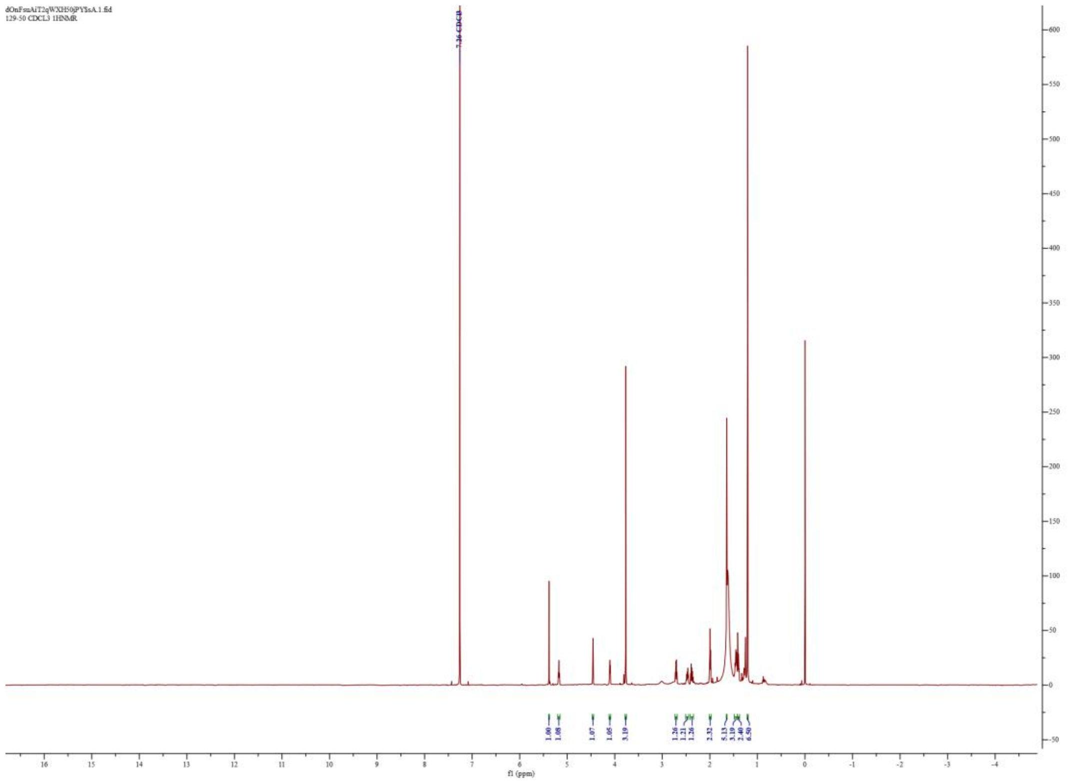

Figure 10S. ^{1}H NMR (600 MHz, $CDCl_3$) spectrum of compound 2.

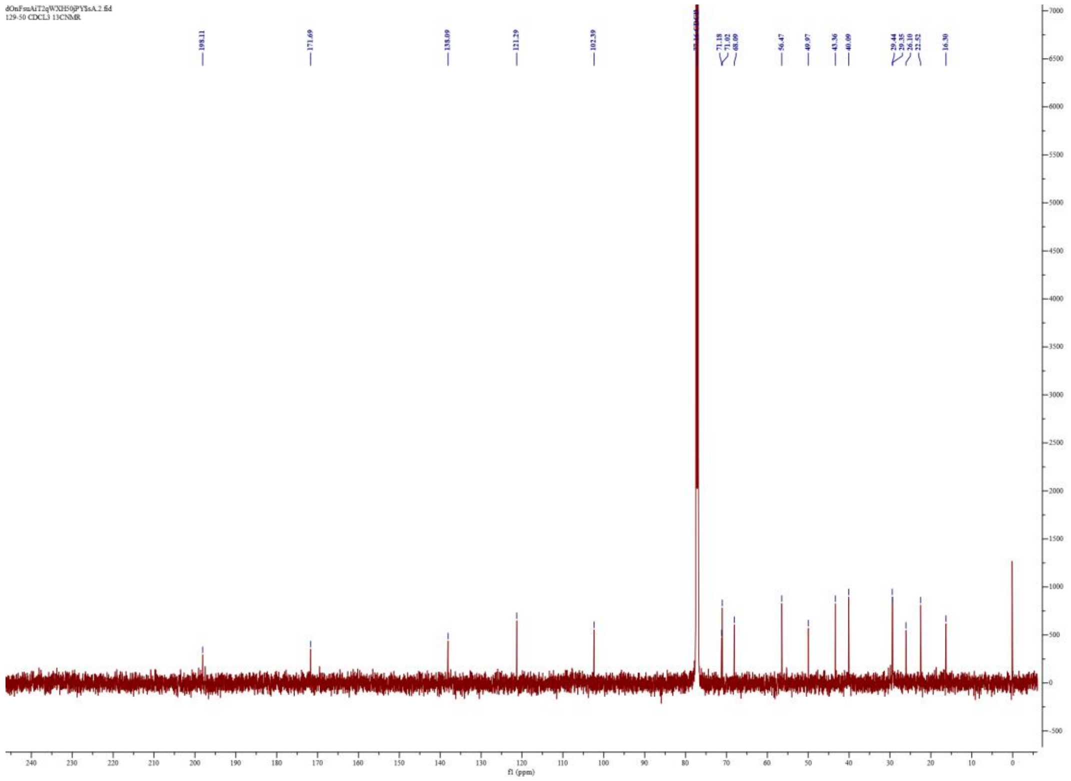

Figure 11S. ^{13}C NMR (125 MHz, $CDCl_3$) spectrum of compound 2.

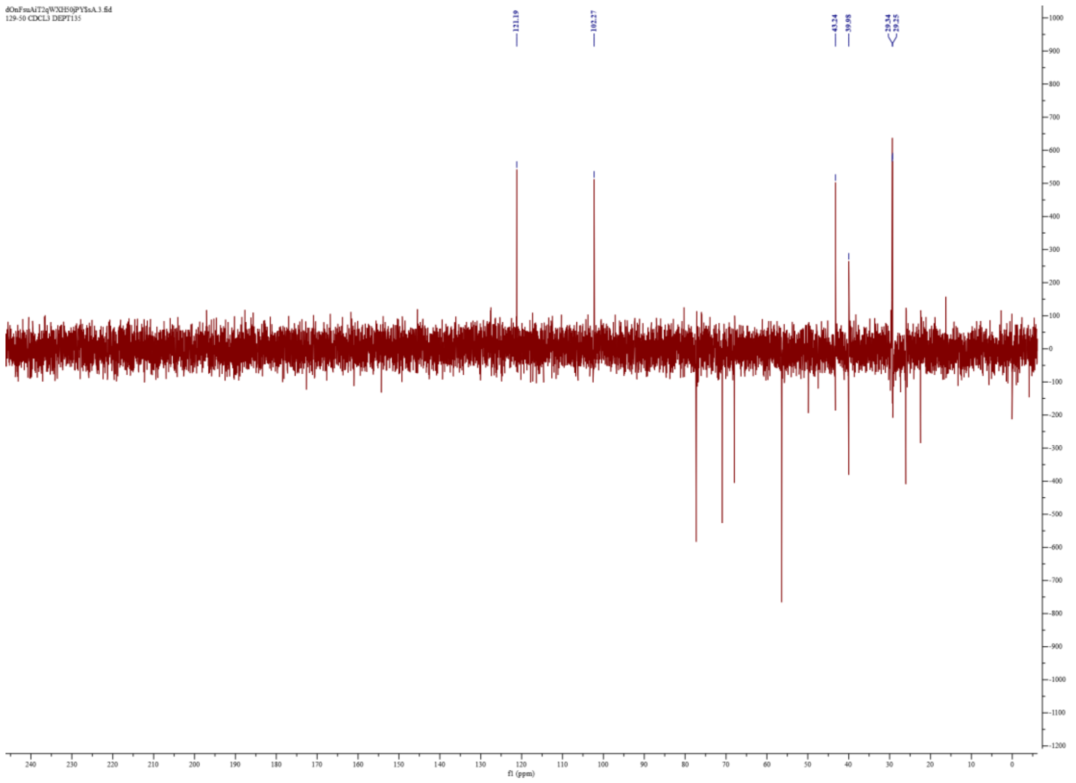

Figure 12S. DEPT spectrum of compound 2.

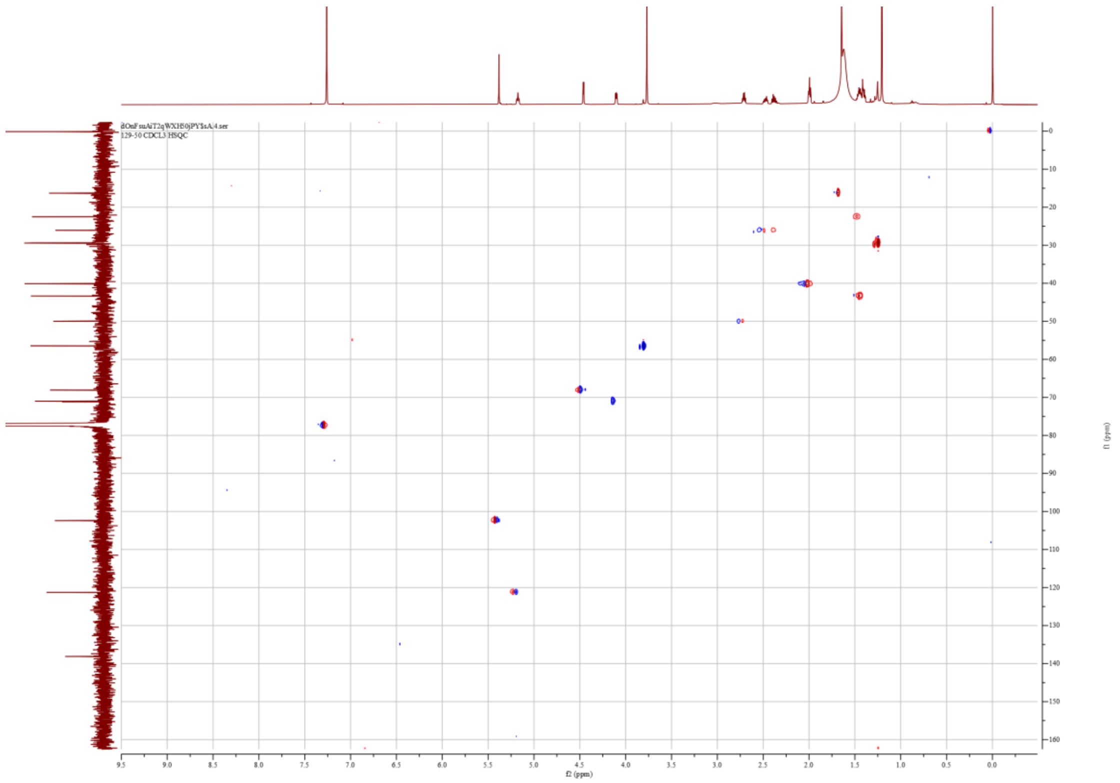

Figure 13S. HSQC spectrum of compound 2.

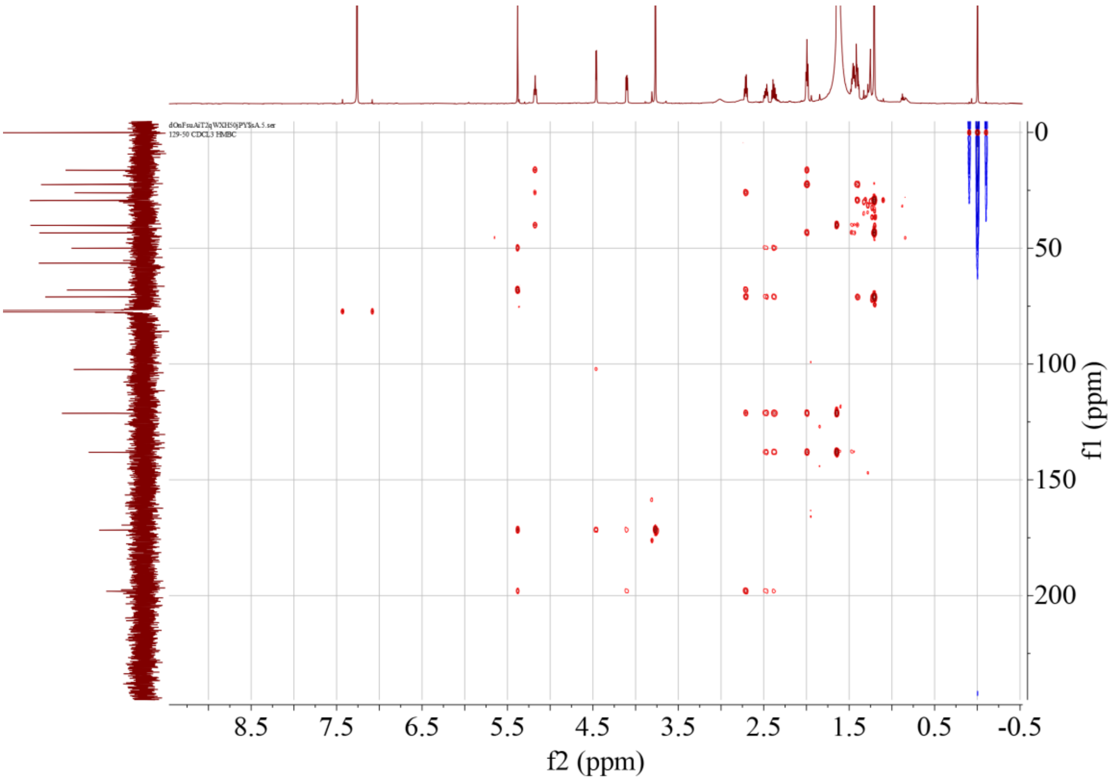

Figure 14S. HMBC spectrum of compound 2.

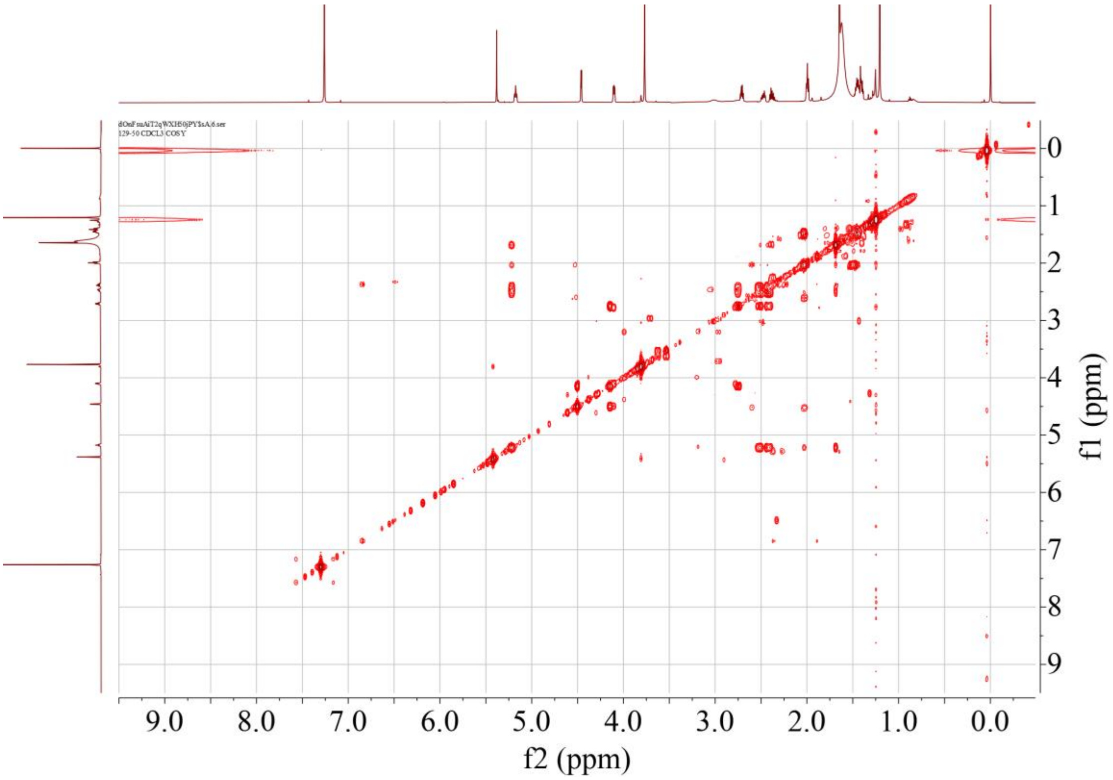

Figure 15S. ^{1}H-^{1}H COSY spectrum of compound 2.

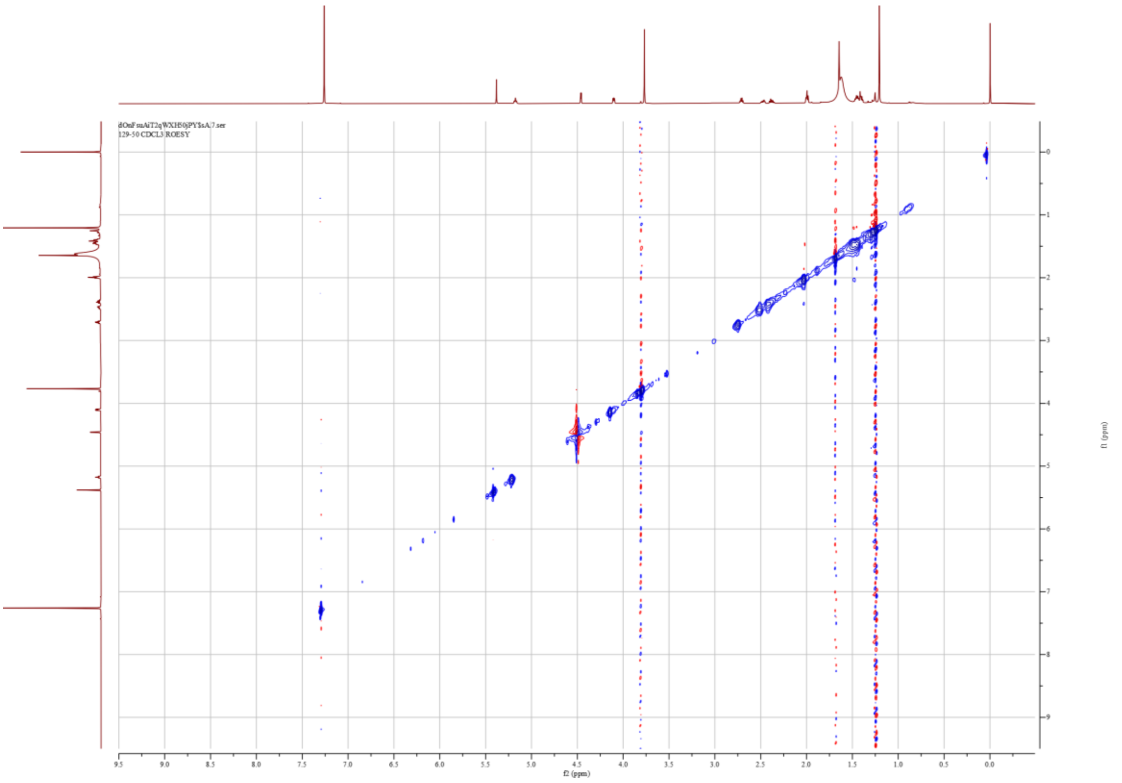

Figure 16S. ROESY spectrum of compound 2.

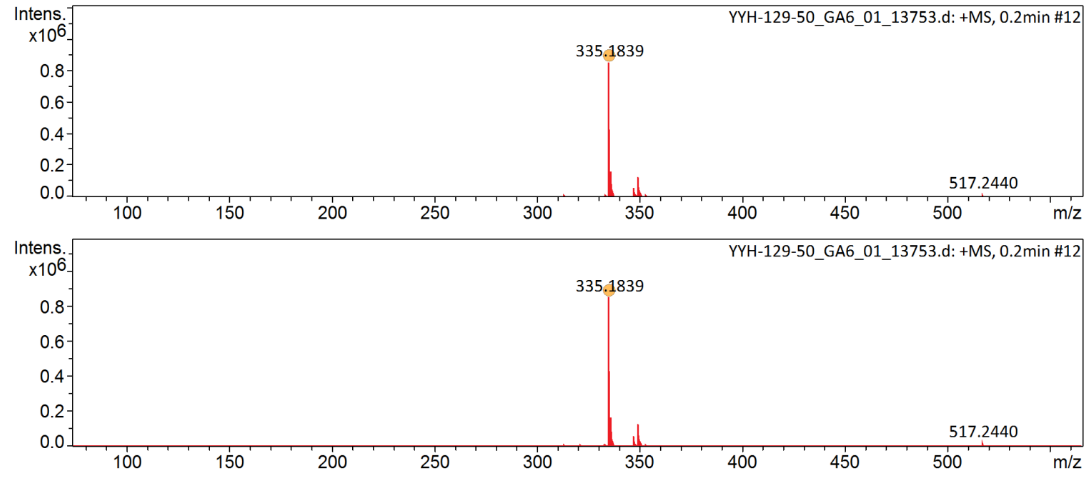

Meas. m/z	#	Ion Formula	m/z	err [ppm]	mSigma	# mSigma	Score	rdb	e¯ Conf	N-Rule	Adduct
335.1839	1	C17H28NaO5	335.1829	-3.1	1.2	1	100.00	4.0	even	ok	M+Na

Figure 17S. HRESIMS spectrum of compound 2.

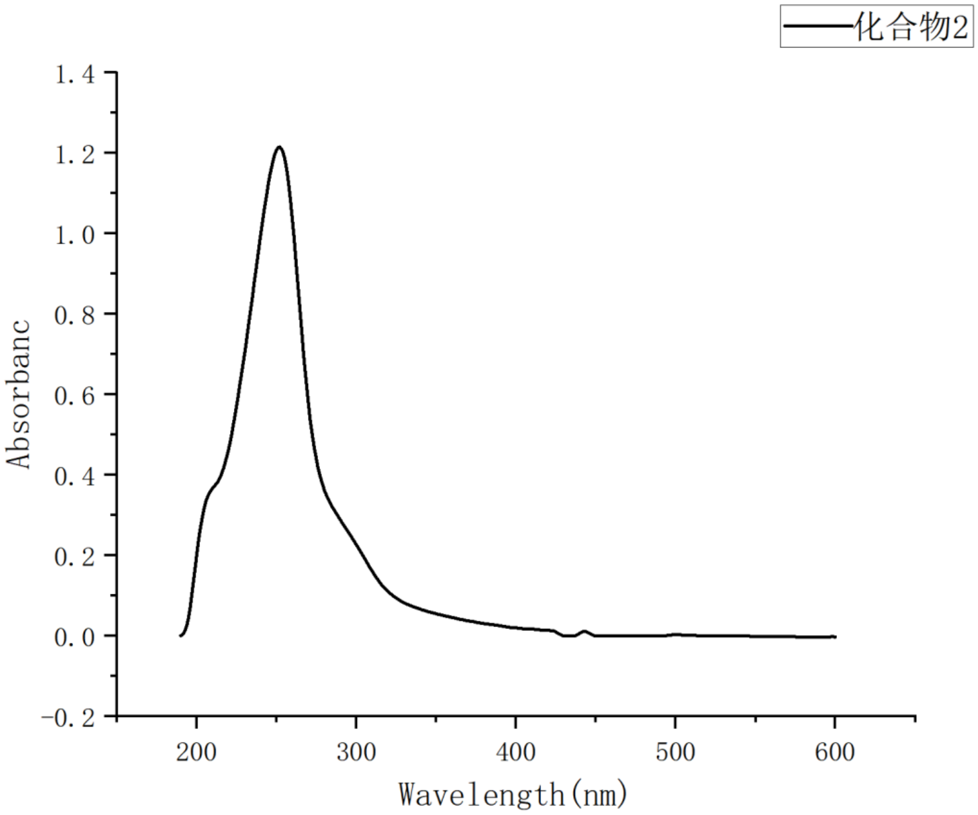

Figure 18S. UV spectrum of compound 2.

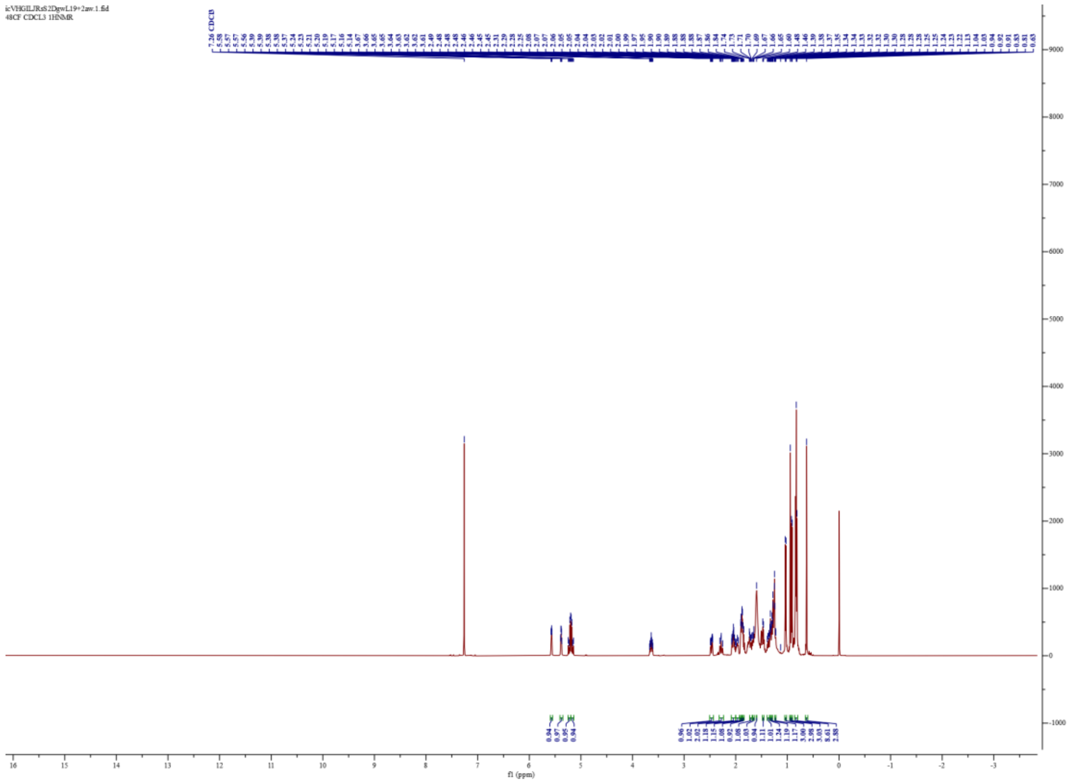

Figure 19S. ^{1}H NMR (600 MHz, $CDCl_3$) spectrum of compound 3.

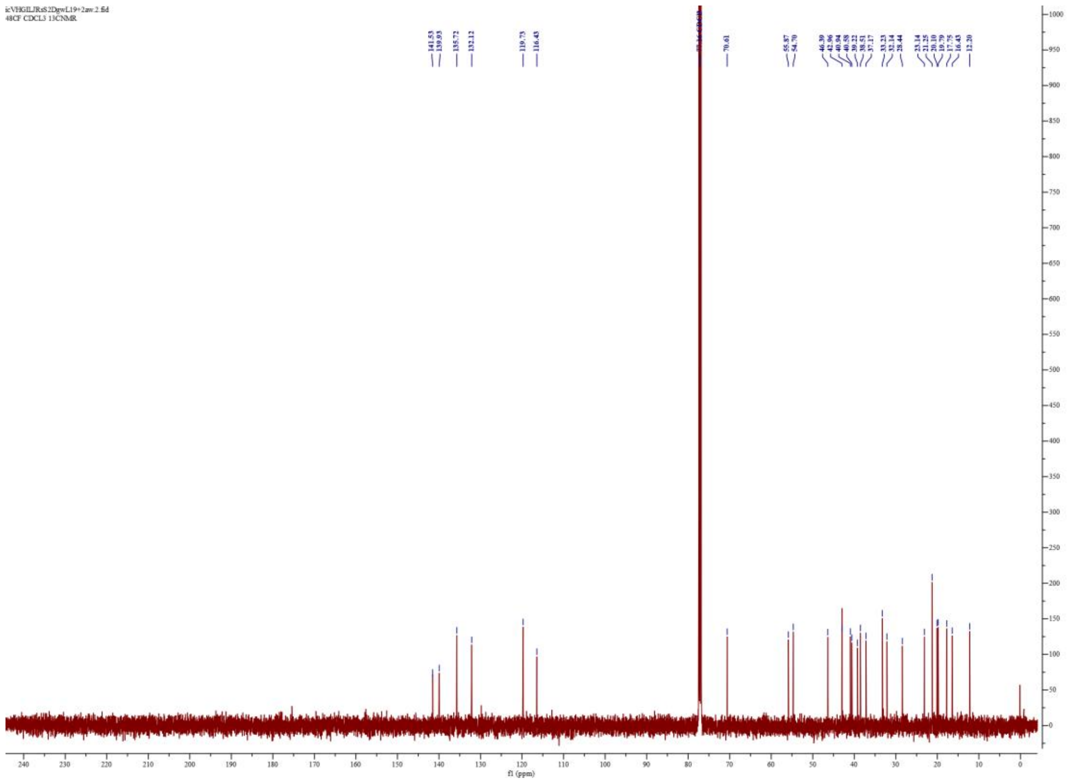

Figure 20S. ^{13}C NMR (125 MHz, $CDCl_3$) spectrum of compound 3.

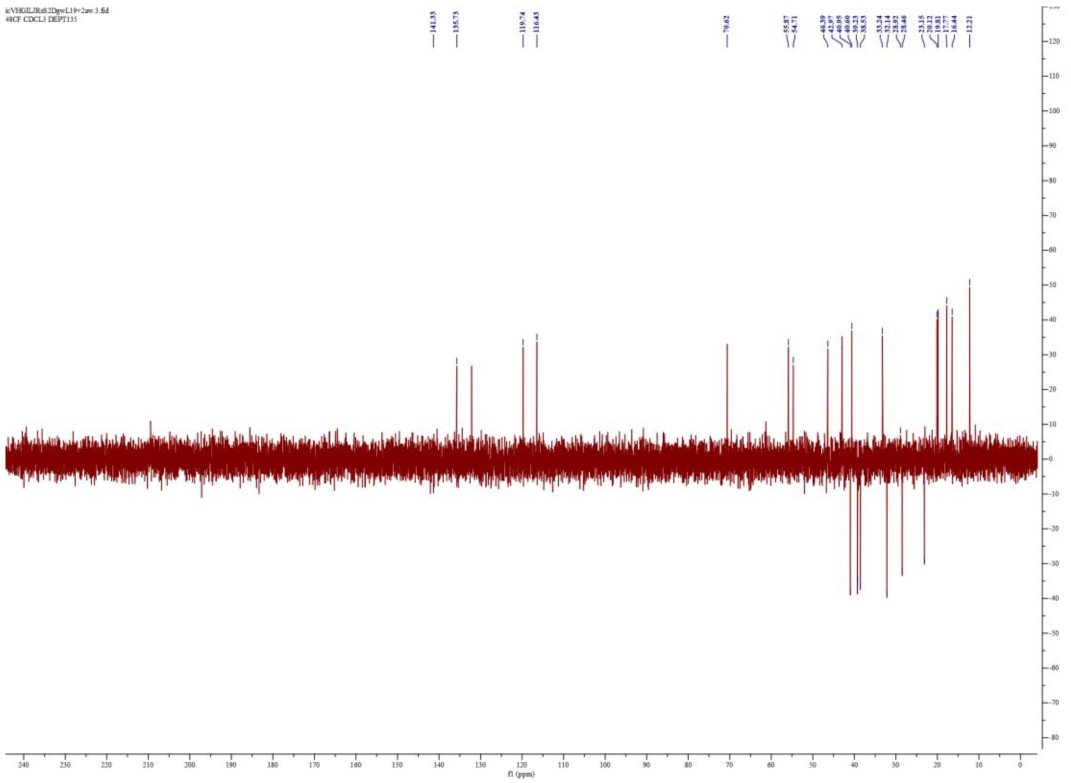

Figure 21S. DEPT spectrum of compound 3.

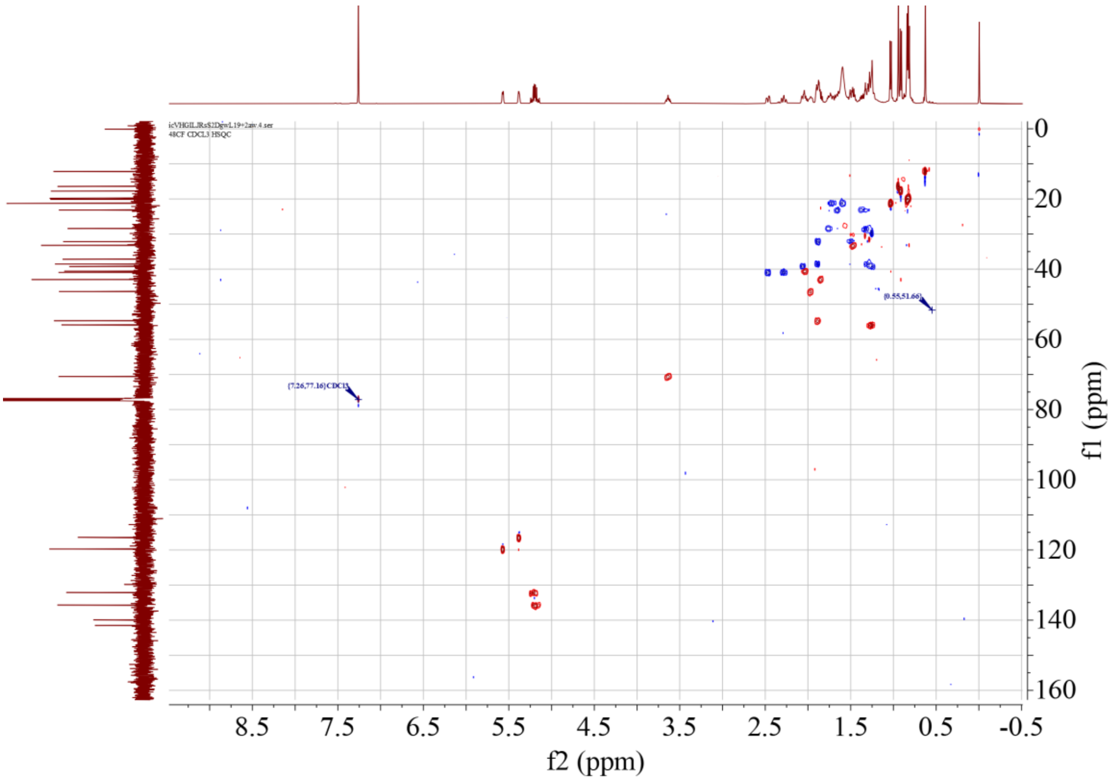

Figure 22S. HSQC spectrum of compound 3.

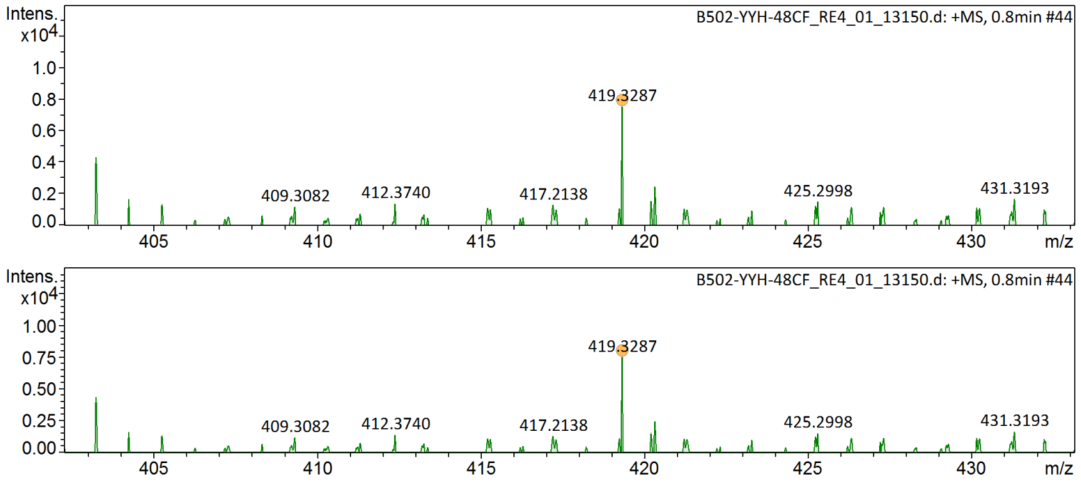

Meas. m/z	#	Ion Formula	m/z	err [ppm]	mSigma	# mSigma	Score	rdb	e⁻ Conf	N-Rule	Adduct
419.3287	1	C28H44NaO	419.3284	-0.7	29.4	1	100.00	7.0	even	ok	M+Na

Figure 23S. HRESIMS spectrum of compound 3.

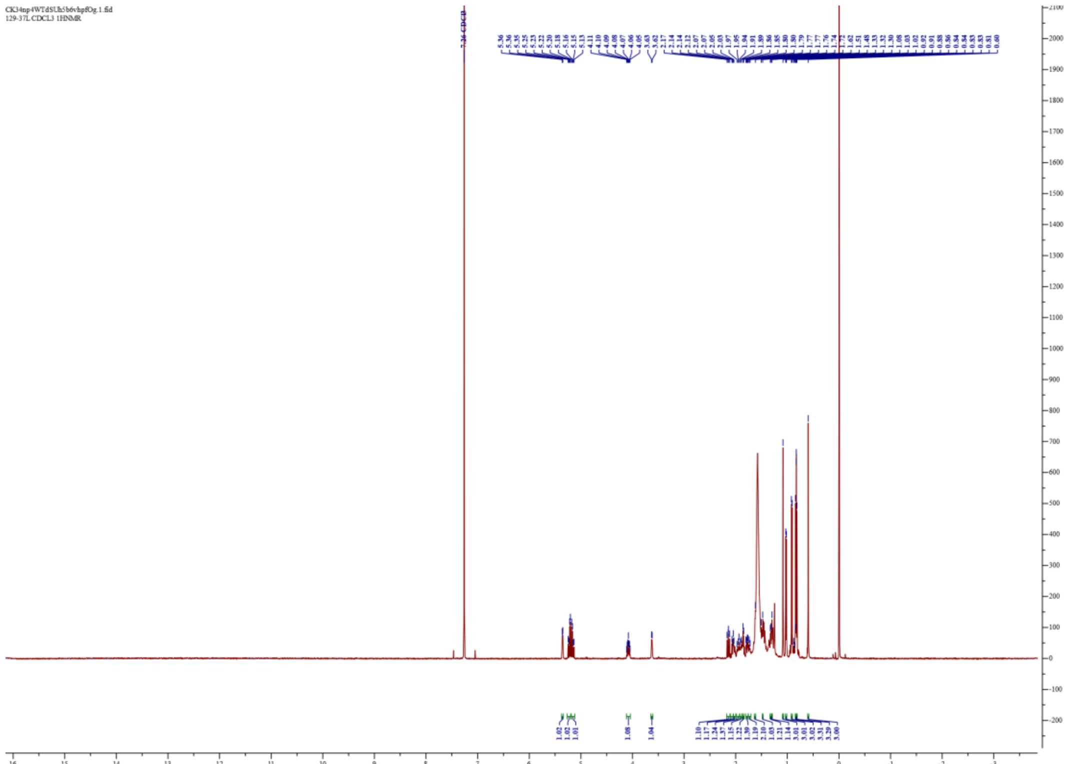

Figure 24S. ^{1H} **NMR (600 MHz, $CDCl_3$) spectrum of compound 4.**

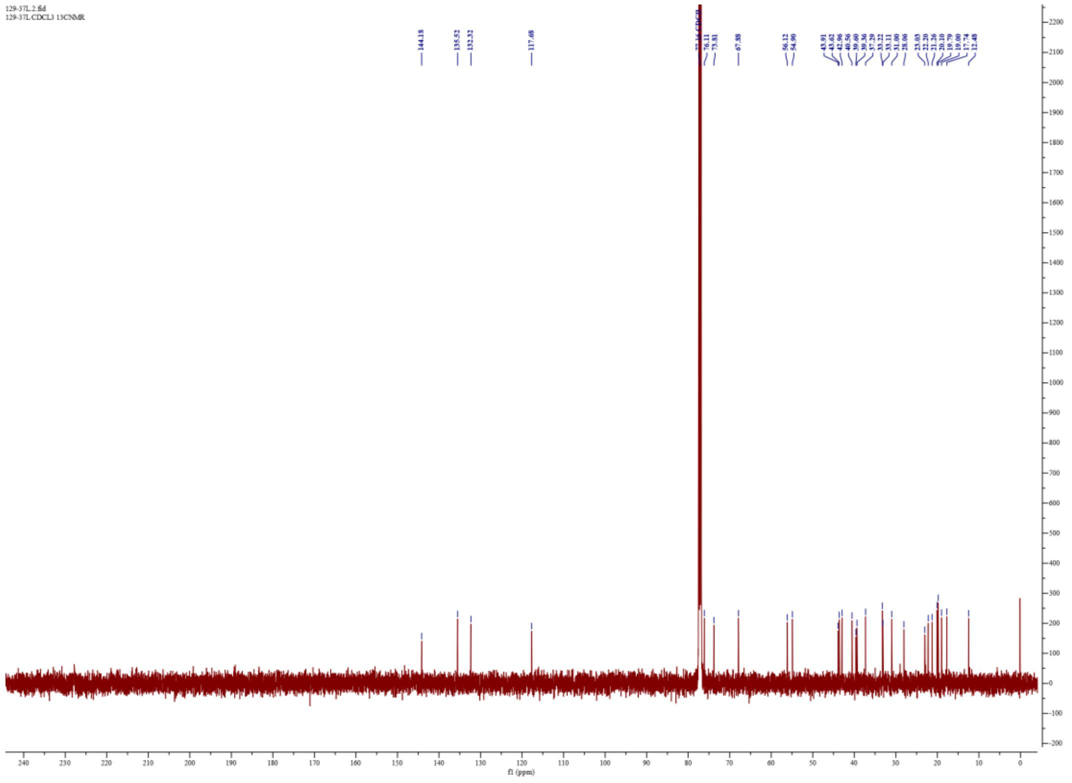

Figure 25S. ^{13}C **NMR (125 MHz, $CDCl_3$) spectrum of compound 4.**

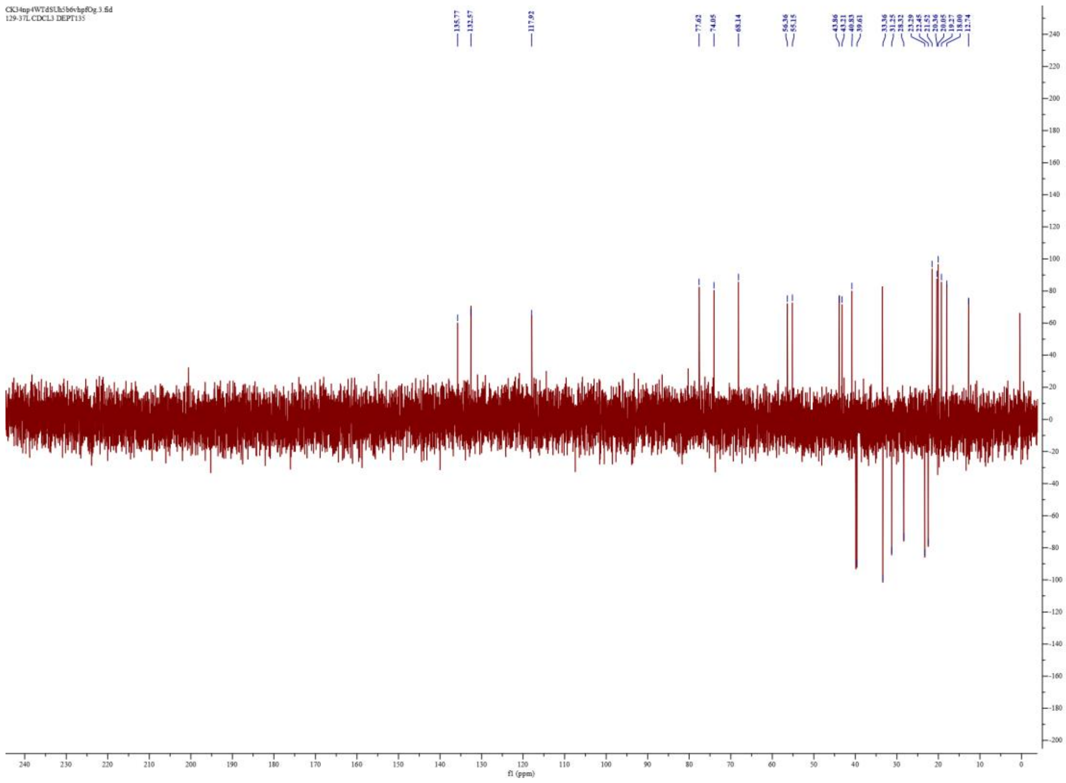

Figure 26S. DEPT spectrum of compound 4.

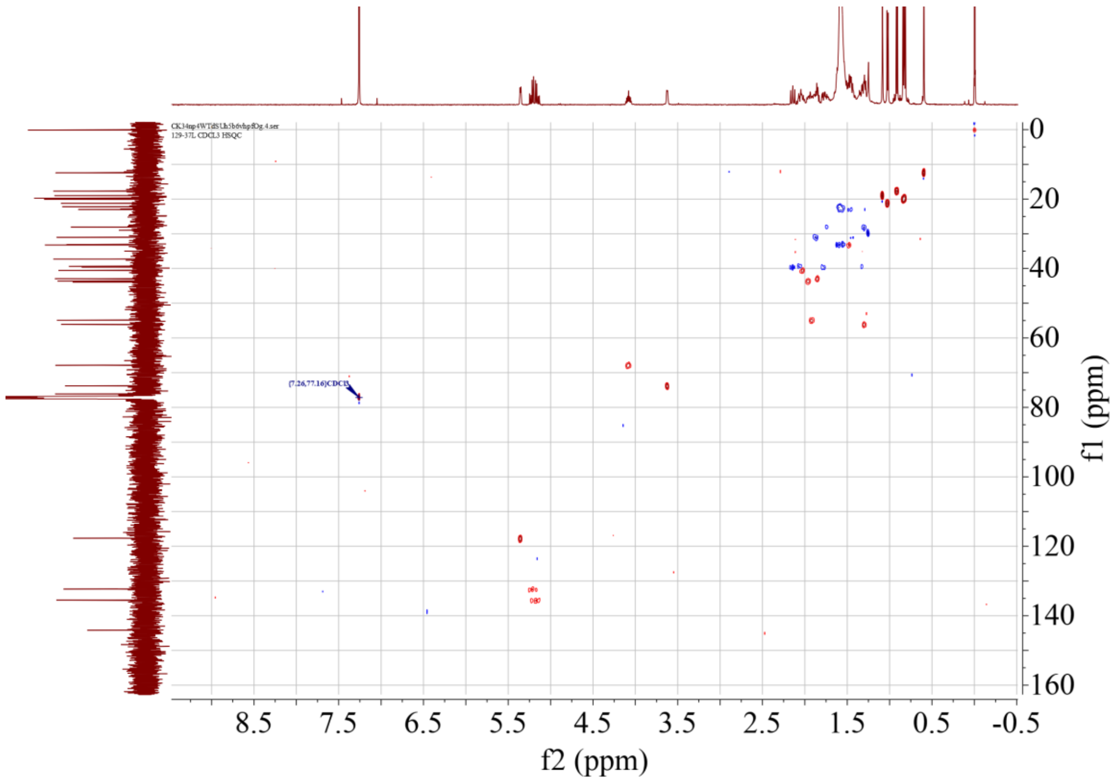

Figure 27S. HSQC spectrum of compound 4.

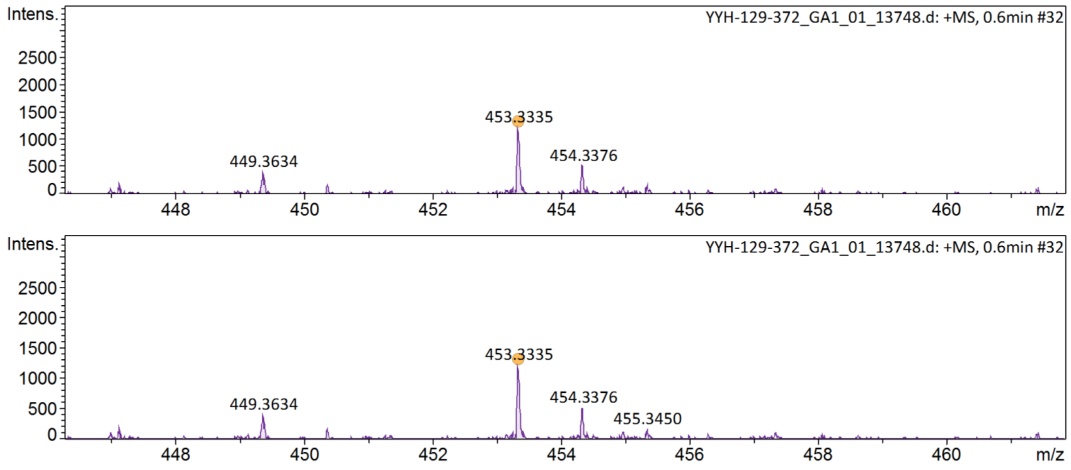

Meas. m/z	#	Ion Formula	m/z	err [ppm]	mSigma	# mSigma	Score	rdb	e¯ Conf	N-Rule	Adduct
453.3335	1	C28H46NaO3	453.3339	0.9	67.6	1	100.00	6.0	even	ok	M+Na

Figure 28S. HRESIMS spectrum of compound 4.

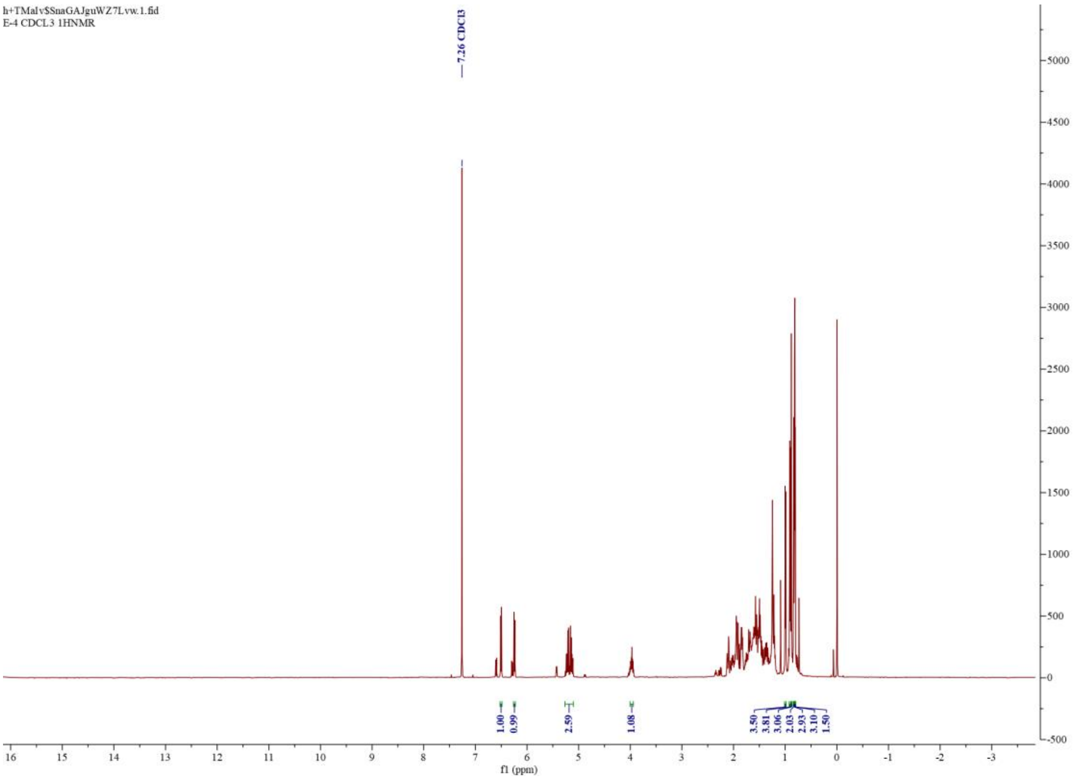

Figure 29S. ^{1}H NMR (600 MHz, $CDCl_3$) spectrum of compound 5.

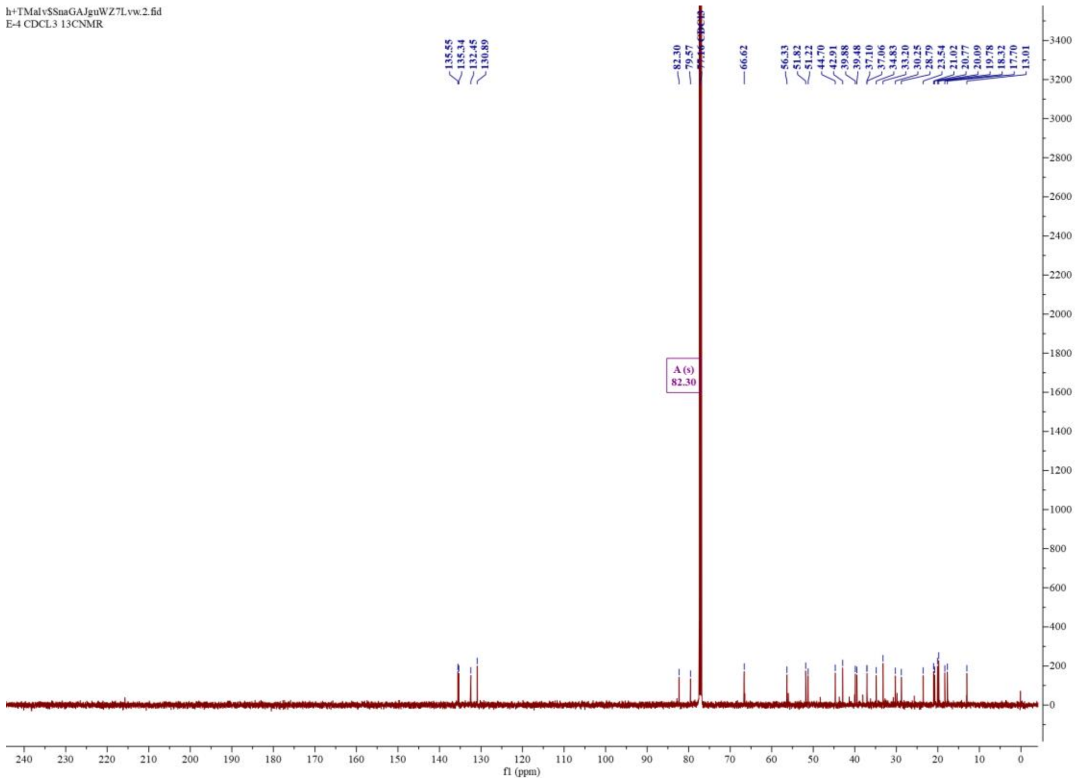

Figure 30S. ^{13}C NMR (125 MHz, $CDCl_3$) spectrum of compound 5.

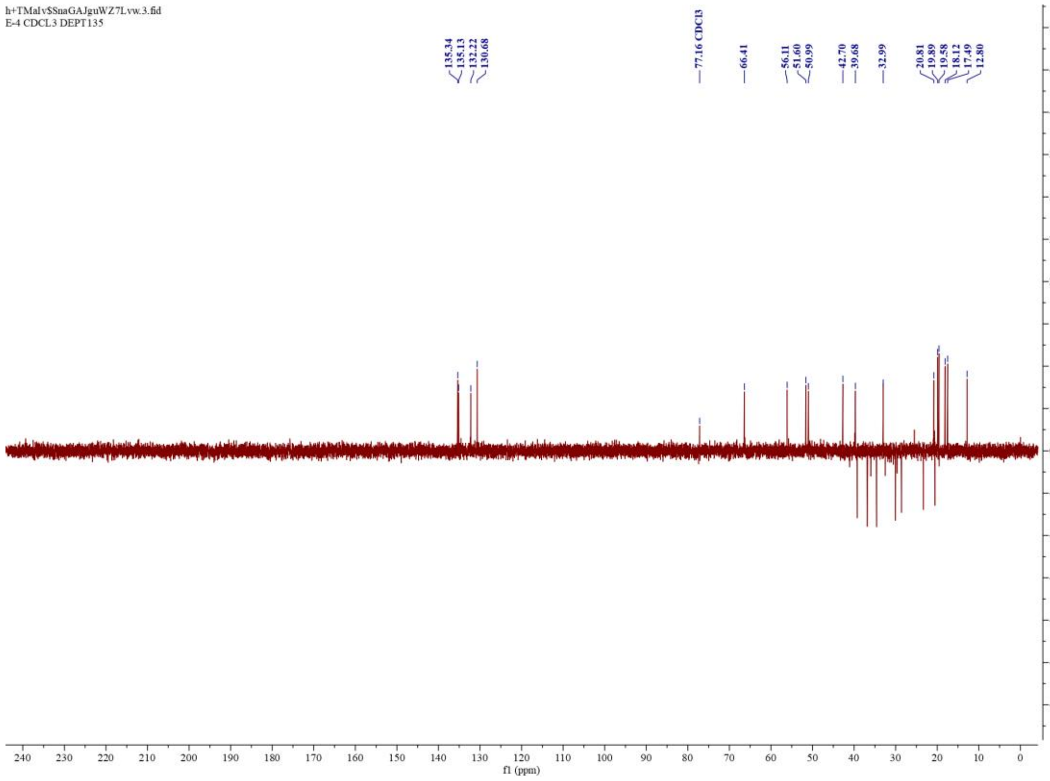

Figure 31S. DEPT spectrum of compound 5.

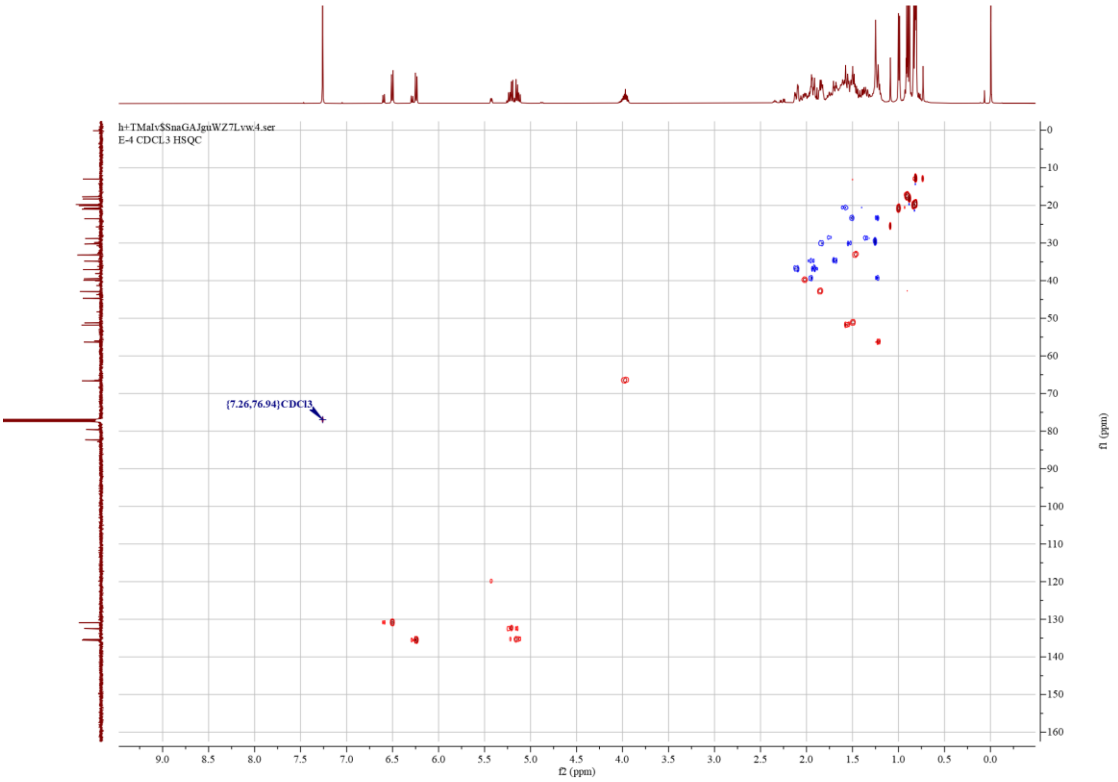

Figure 32S. HSQC spectrum of compound 5.

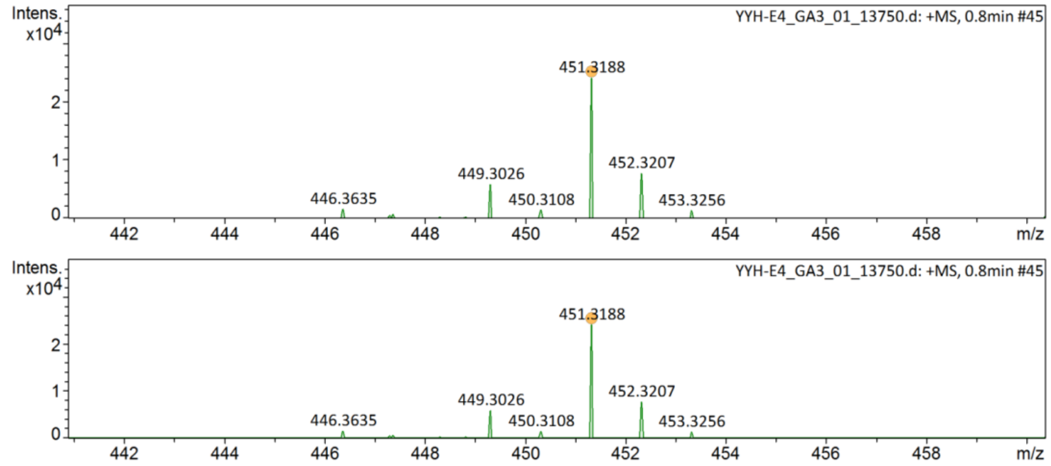

Meas. m/z	#	Ion Formula	m/z	err [ppm]	mSigma	# mSigma	Score	rdb	e⁻ Conf	N-Rule	Adduct
451.3188	1	C28H44NaO3	451.3183	-1.1	6.6	1	100.00	7.0	even	ok	M+Na

Figure 33S. HRESIMS spectrum of compound 5.

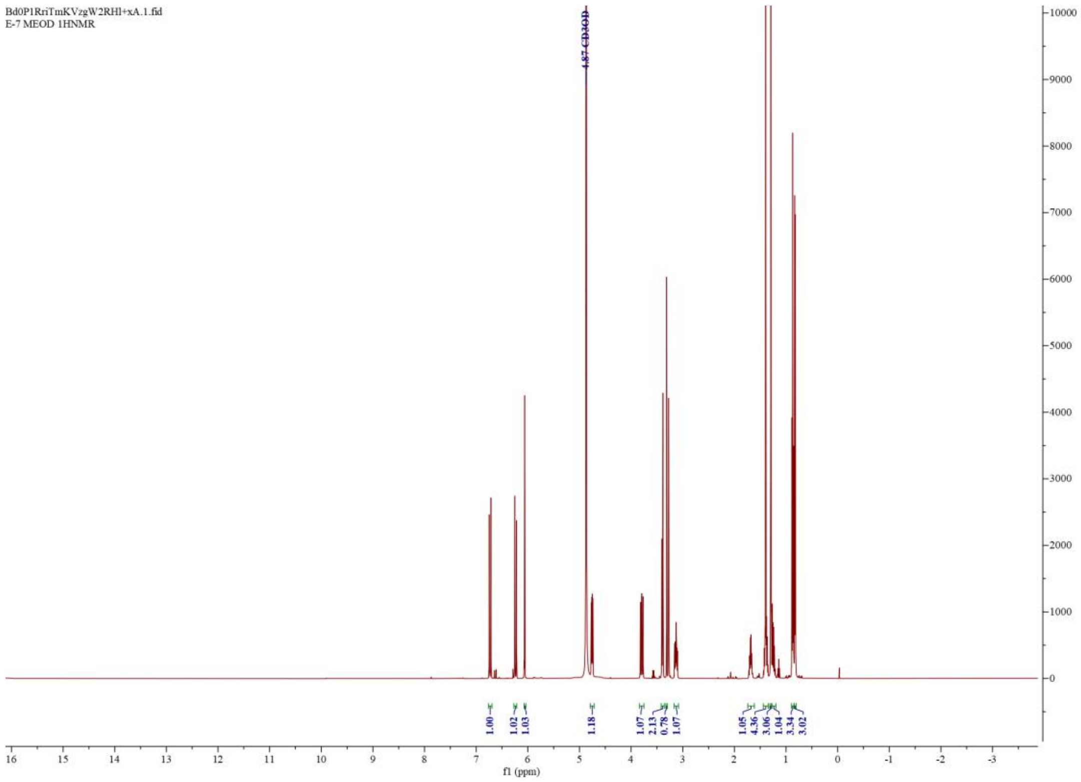

Figure 34S. ^{1}H **NMR (600 MHz, MeOD) spectrum of compound 6.**

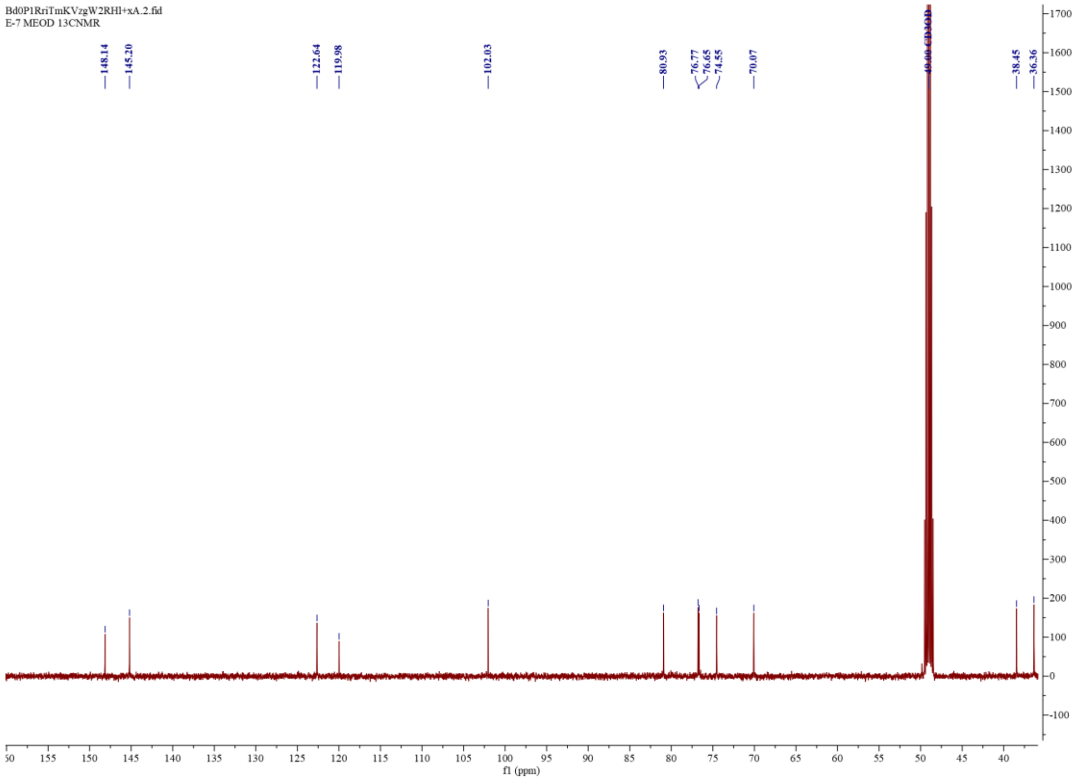

Figure 35S. ^{13}C **NMR (125 MHz, MeOD) spectrum of compound 6.**

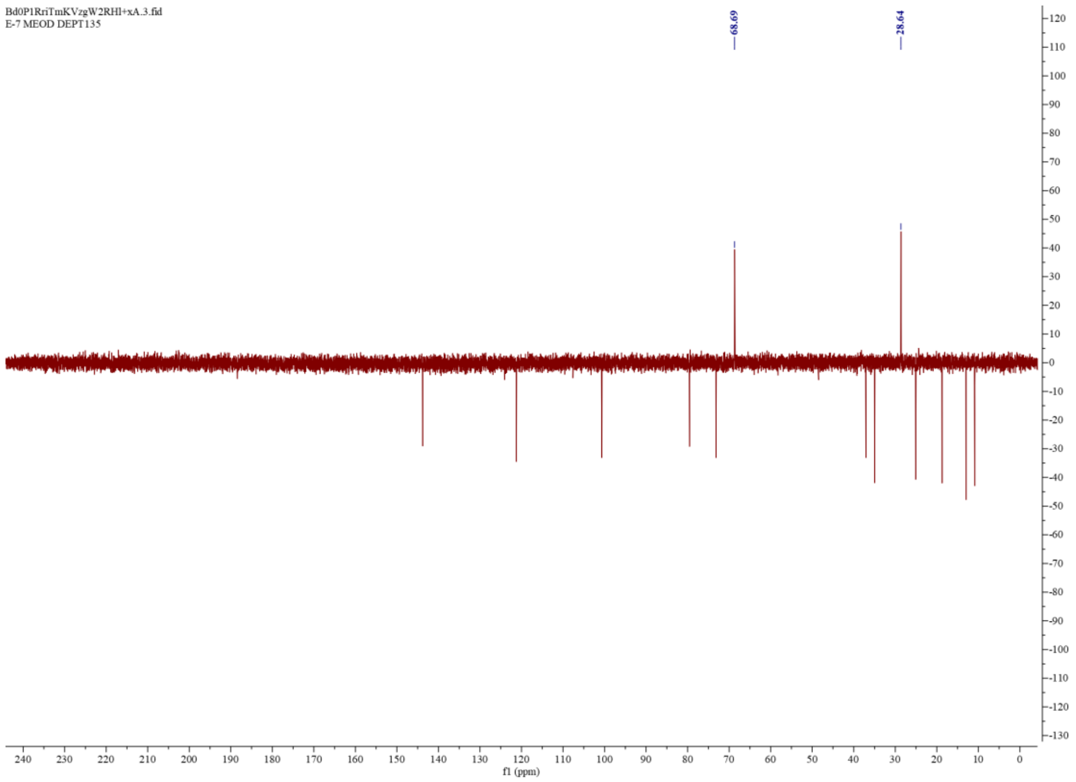

Figure 36S. DEPT spectrum of compound 6.

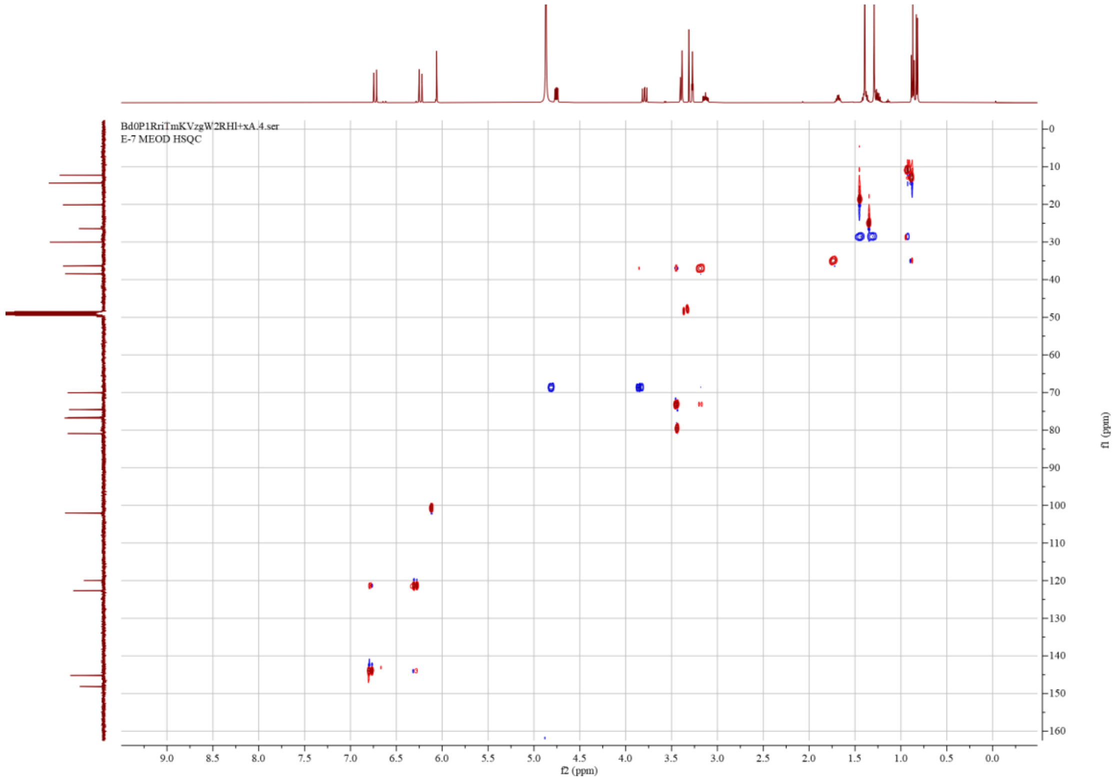

Figure 37S. HSQC spectrum of compound 6.

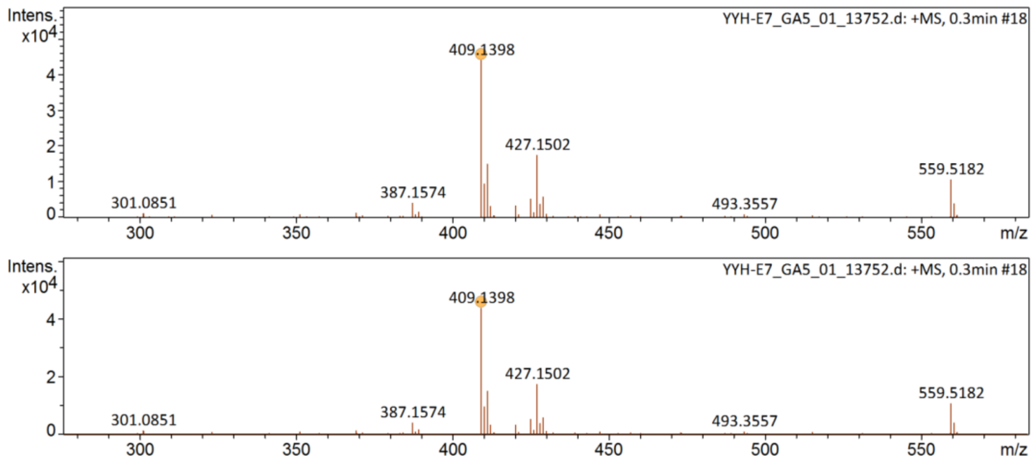

Meas. m/z	#	Ion Formula	m/z	err [ppm]	mSigma	# mSigma	Score	rdb	e¯ Conf	N-Rule	Adduct
409.1398	1	C19H27ClNaO6	409.1388	-2.5	6.4	1	100.00	6.0	even	ok	M+Na

Figure 38S. HRESIMS spectrum of compound 6.

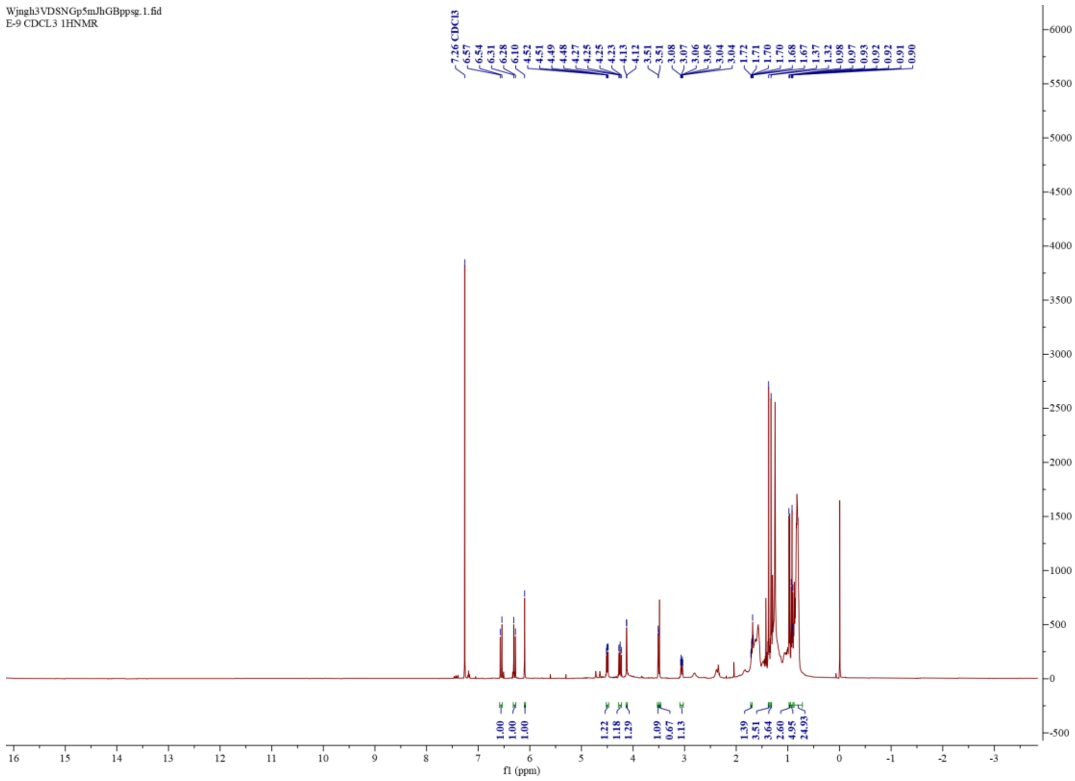

Figure 39S. 1H NMR (600 MHz, $CDCl_3$) spectrum of compound 7.

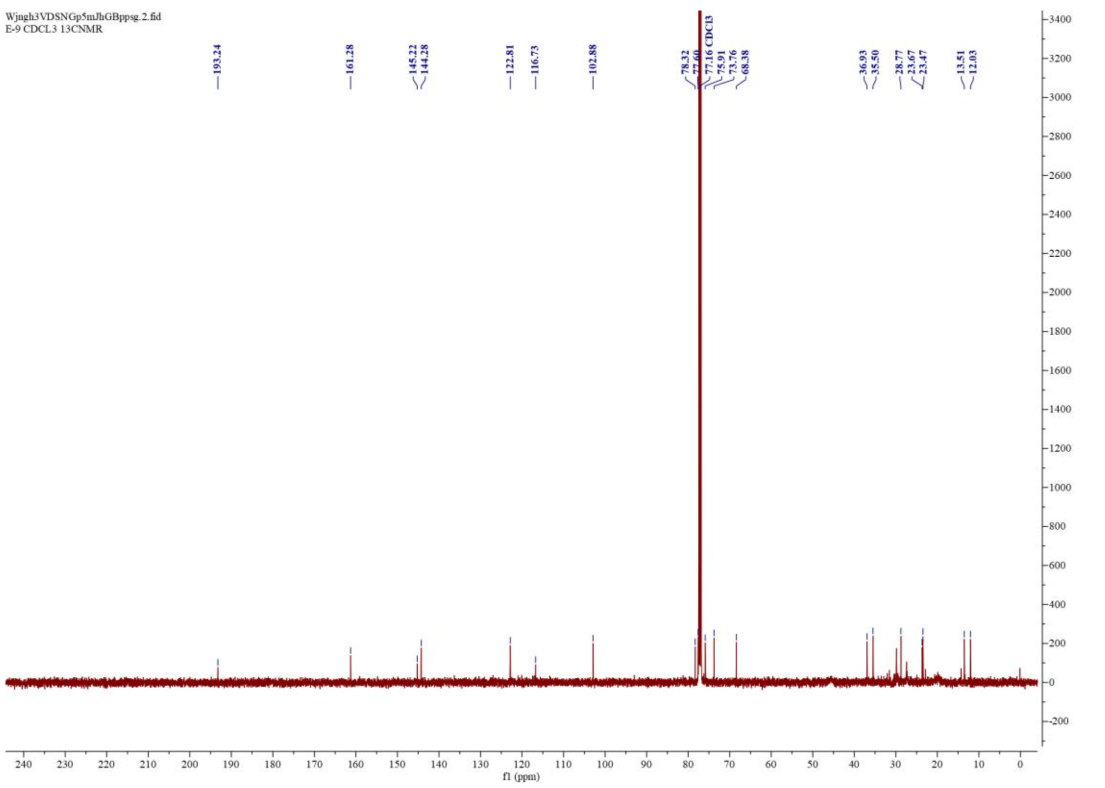

Figure 40S. ^{13}C NMR (125 MHz, $CDCl_3$) spectrum of compound 7.

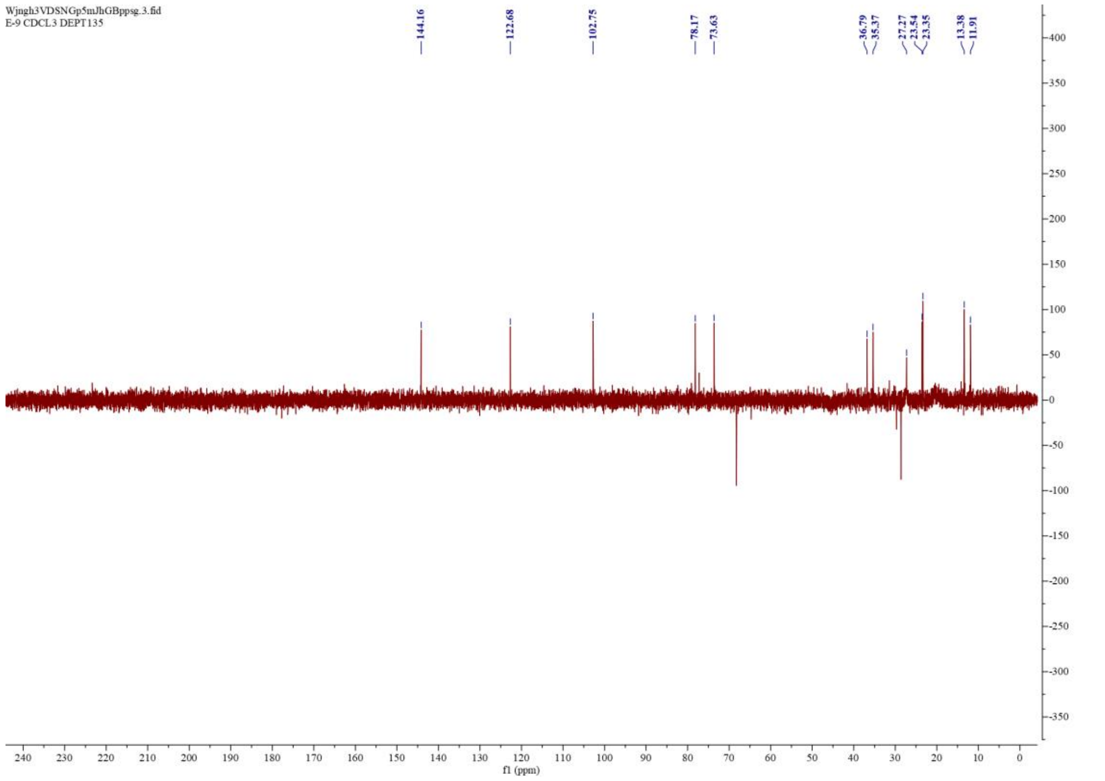

Figure 41S. DEPT spectrum of compound 7.

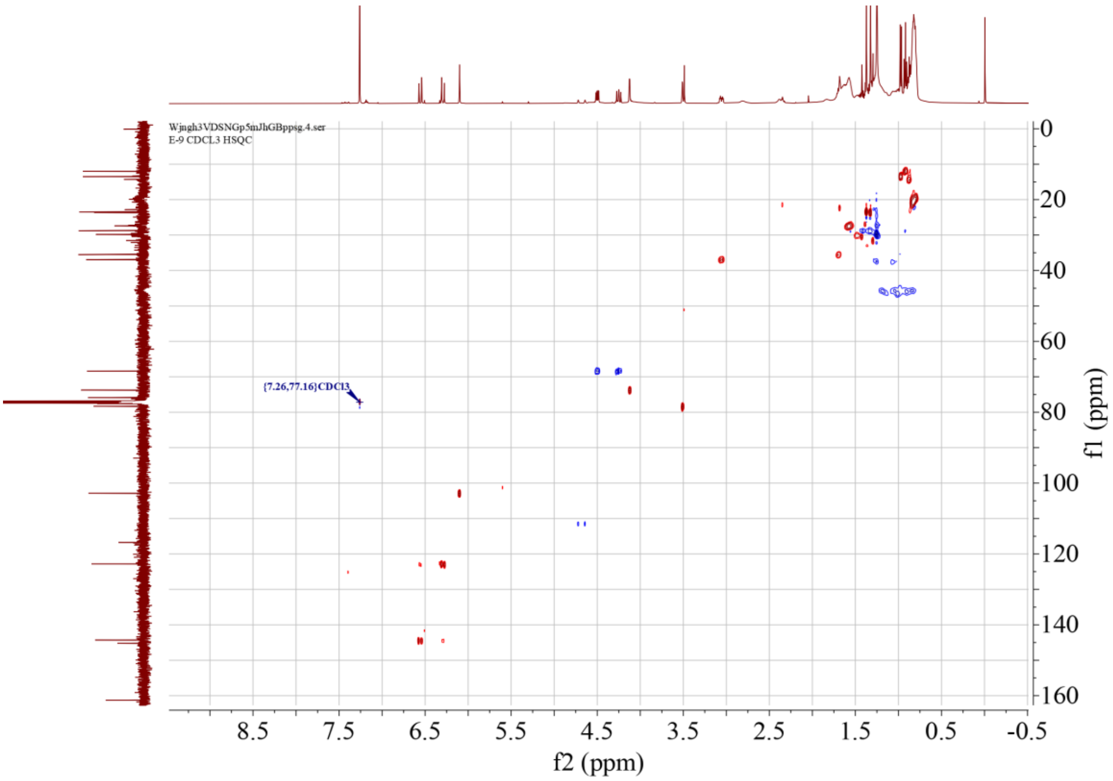

Figure 42S. HSQC spectrum of compound 7.

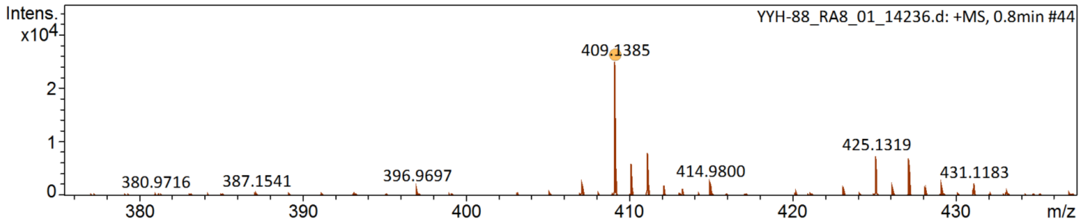

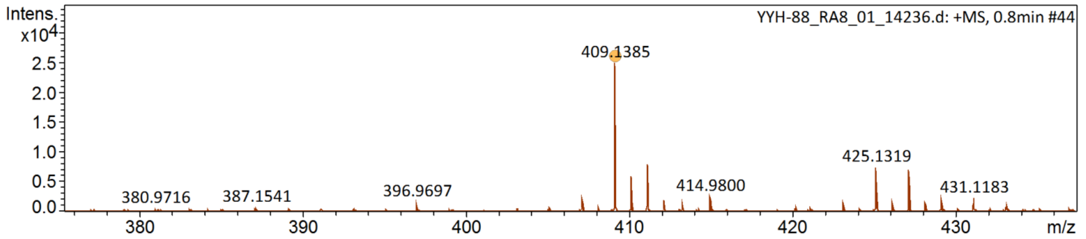

Meas. m/z	#	Ion Formula	m/z	err [ppm]	mSigma	# mSigma	Score	rdb	e⁻ Conf	N-Rule	Adduct
409.1385	1	C19H27ClNaO6	409.1388	0.8	20.2	1	100.00	6.0	even	ok	M+Na

Figure 43S. HRESIMS spectrum of compound 7.
